# Self-Assembling Comb-like Fluorinated Epoxy Acrylates for Low Surface Energy UV-Curable Coatings

**DOI:** 10.3390/polym18182259

**Published:** 2026-09-16

**Authors:** Yejung Lee, Hyesu Jang, Daeun Jeong, Su Bin Ji, Ye Rim Cho, Namhee Kim, Myung Hwa Kim, Yong-Ju Kwon

**Affiliations:** 1 Department of Chemistry, Ewha Womans University, Seoul 03760, Republic of Korea; ylee682@sas.upenn.edu (Y.L.); nam222ee@ewha.ac.kr (N.K.); myungkim@ewha.ac.kr (M.H.K.); 2 Department of Chemistry, Seoul Women’s University, Seoul 01797, Republic of Korea; jdaeun2404@gmail.com (D.J.); wltnqls324@swu.ac.kr (S.B.J.); esther1218@swu.ac.kr (Y.R.C.)

**Keywords:** photopolymerization, urethane acrylate, surface segregation, fluorine efficiency, water contact angle, hydrophobic coating, perfluorophenyl

## Abstract

Fluorinated UV-curable coatings typically rely on high fluorine loadings to achieve water repellency, which causes phase separation, high cost, and environmental concerns. Here, a novel UV-curable fluorinated epoxy acrylate (FEA) oligomer with a comb-like architecture bearing perfluorophenyl-terminated fluorinated side chains was synthesized to maximize fluorine efficiency. A mono-substituted fluorinated alcohol was prepared via nucleophilic aromatic substitution of hexafluorobenzene with octafluorohexanediol, and the FEA oligomer was constructed through a two-step urethane-forming reaction with diglycerol diacrylate (DGDA) and isophorone diisocyanate (IPDI). The chemical structures were confirmed by FT-IR and ^1^H/^19^F NMR spectroscopy. UV-cured FEA/HDDA-F films exhibited high double-bond conversions of 93.5–97.9% and adequate thermal stability for coating applications. As the FEA content increased, the advancing water contact angle rose from 69° to 118°, and the surface free energy decreased from 34.0 to 19.2 mJ/m^2^, comparable to that of polytetrafluoroethylene (18.5 mJ/m^2^). Remarkably, this performance was achieved at a lower overall fluorine content than the fluorinated diluent alone, and the near-zero polar component of the surface energy, together with XPS analysis and SEM-observed surface texturing, supports the proposed preferential segregation of the fluorinated side chains toward the air–film interface. The comb-like FEA is a promising fluorine-efficient material for UV-curable hydrophobic and self-cleaning coatings.

## 1. Introduction

Surface modification of polymeric materials has become a vital research area because of its wide range of industrial demands for self-cleaning, anti-fouling, and water-repellent coatings and films [1,2]. Among the available fabrication techniques, UV-curing technology has emerged as a highly efficient method owing to its rapid polymerization rate, low energy consumption, solvent-free processability, and negligible emission of volatile organic compounds (VOCs) [3,4]. UV-curable formulations based on acrylate chemistry are particularly attractive, as the free-radical polymerization of acrylate groups proceeds within seconds under ambient conditions and yields films with good optical clarity and adhesion [5]. A typical UV-curable formulation comprises three components: an oligomer, which forms the backbone of the network and governs the mechanical, thermal, and surface properties of the cured film; a reactive diluent, a low-molecular-weight monomer that lowers the viscosity while being covalently incorporated into the network; and a photoinitiator, which generates the initiating species upon irradiation. Curing proceeds either by free-radical polymerization of (meth)acrylate groups, which is fast but susceptible to oxygen inhibition, or by cationic ring-opening polymerization of epoxides and vinyl ethers, which is insensitive to oxygen but requires humidity control. Such formulations are widely used in protective coatings, printing inks, adhesives, optical films, and negative-tone photoresists, where the attainable resolution is limited by oxygen inhibition, light scattering, and cure shrinkage [3,4,5]. Among acrylate oligomers, epoxy acrylates—prepared by end-capping epoxy resins with acrylate groups through epoxide ring-opening—are widely employed because the ring-opening reaction generates pendant hydroxyl groups along the backbone, which not only enhance adhesion but also serve as versatile sites for further functionalization [6,7].

Incorporating fluorine into UV-curable coatings is a well-established strategy for imparting hydrophobicity, low surface tension, low refractive index, and outstanding chemical and thermal inertness, all of which originate from the strong electronegativity, small ionic radius, and low polarizability of the fluorine atom [8,9]. However, conventional approaches that simply increase the bulk fluorine content to improve hydrophobicity face practical limitations. High loadings of fluorinated segments cause significant phase separation and poor compatibility with the polymer matrix, degrading the mechanical integrity, optical clarity, and coating uniformity of the resulting films [10,11]. In addition, fluorinated raw materials are costly, and growing environmental regulations on long-chain perfluoroalkyl substances have intensified the demand for coating systems that achieve high water repellency with a minimal amount of short-chain fluorinated segments [12,13]. Achieving high “fluorine efficiency”—that is, maximizing surface performance per unit of fluorine incorporated—therefore remains a central challenge in the design of fluorinated UV-curable coatings.

A promising solution is a molecular architecture that drives the selective enrichment of fluorinated segments at the coating–air interface rather than their homogeneous distribution in the bulk. In comb-like fluorinated oligomers, fluorinated side chains covalently attached to a UV-curable backbone can migrate and orient toward the air–polymer interface during film formation, so that a thin fluorinated surface layer governs the wetting behavior while the bulk properties of the film are preserved [14,15,16]. Although several comb-type fluorinated acrylates have been reported, most previous systems rely on aliphatic (per)fluoroalkyl side chains grafted onto aliphatic backbones [17,18,19]; comb-like epoxy acrylates bearing an aromatic perfluorophenyl terminal group on each side chain have rarely been explored, despite the potential of the rigid, highly fluorinated aromatic ring to further lower the surface energy and to provide additional thermal and chemical robustness. Moreover, a systematic demonstration that such an architecture can outperform conventional fluorinated diluents even at a lower overall fluorine content is still lacking. Beyond conventional fluorinated acrylates, recent approaches to low-surface-energy materials increasingly rely on controlling how functional groups organize at the interface rather than on their bulk concentration, as seen in surface-segregating polymer systems [14,15,16]. The comb-like architecture pursued here belongs to this family of interface-engineering strategies, in which the spatial placement of the fluorinated segment rather than its overall loading governs the surface performance. Recent comb-like coating systems have confirmed that the placement of functional side chains, rather than their bulk loading, governs interfacial behavior [20,21]. These systems, however, rely on crystalline alkyl or aliphatic perfluoroalkyl pendant groups on waterborne polyurethane backbones, whereas the present FEA oligomer employs a rigid perfluoroaromatic terminus within a solvent-free UV-curable acrylate network.

Recent lubricant-inspired polymer coatings have combined self-lubricating surface layers with embedded silicon-oil reservoirs to reduce ice adhesion [22]. Alternatively, covalently attached, mobile polysiloxane brushes can create liquid-like interfaces that reduce unwanted adhesion [23]. The present comb-like FEA design instead targets low surface energy through the proposed surface segregation of covalently incorporated fluorinated side chains in a UV-cured network, with fluorine efficiency rather than self-lubrication as the central objective.

**Figure 1:**
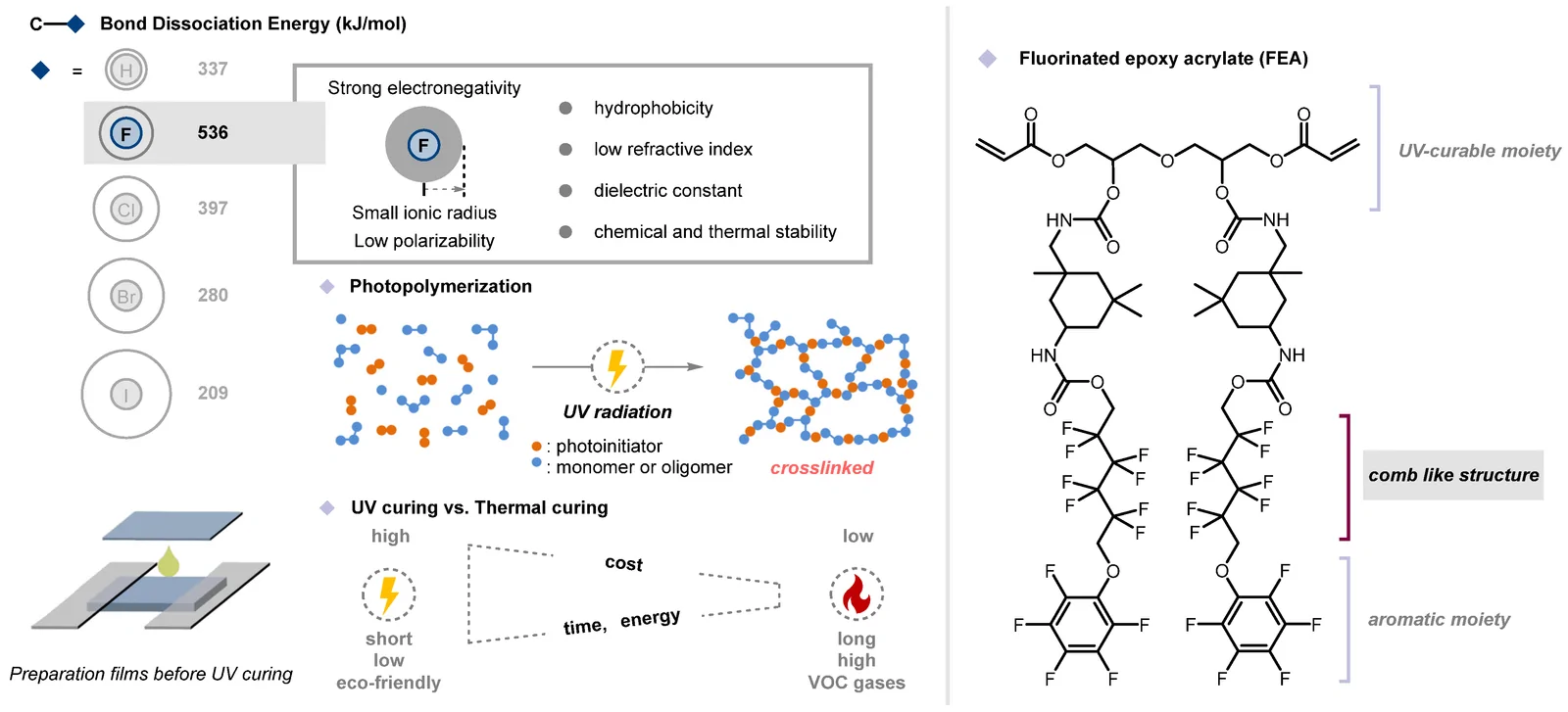
Molecular design concept of self-assembling fluorinated epoxy acrylate coatings.

In this study, we synthesized a novel UV-curable fluorinated epoxy acrylate (FEA) oligomer with a well-defined comb-like structure terminated with perfluorophenyl groups, as illustrated in Figure 1. A mono-substituted fluorinated alcohol was first prepared by the nucleophilic aromatic substitution (SNAr) of hexafluorobenzene with 2,2,3,3,4,4,5,5-octafluorohexane-1,6-diol, and the FEA oligomer was then constructed through a controlled two-step urethane-forming reaction of diglycerol diacrylate (DGDA) with isophorone diisocyanate (IPDI), followed by grafting of the fluorinated alcohol onto the remaining isocyanate groups. The chemical structure was confirmed by FT-IR and ^1^H/^19^F NMR spectroscopy. UV-cured films were formulated by blending the FEA oligomer with a fluorinated reactive diluent (HDDA-F) at various ratios, and their photopolymerization behavior, thermal stability, UV resistance, surface chemical composition, surface morphology, and wetting properties were systematically evaluated. Notably, although the FEA-rich films contained a lower overall fluorine content than the diluent-rich films, they exhibited markedly higher hydrophobicity: the advancing water contact angle increased from 69° to 118°, and the surface free energy decreased to 19.2 mJ/m^2^, comparable to that of polytetrafluoroethylene (PTFE, 18.5 mJ m^−2^). These results demonstrate that the comb-like architecture with perfluoroaromatic end groups enables fluorine-efficient, low-surface-energy coatings and highlight the potential of the FEA oligomer for advanced UV-curable coating applications such as paints, adhesives, and protective films.

## 2. Materials and Methods

### 2.1. Materials

Potassium carbonate anhydrous (K_2_CO_3_, extra pure grade), isopropyl alcohol (IPA, extra pure grade), and hydrochloric acid (HCl, >35.0%) were purchased from Duksan Pure Chemicals Co., Ltd. (Ansan-si, Republic of Korea). *N*,*N*-Dimethylacetamide (DMAc, anhydrous, 99.8%), butyl acetate (BA, anhydrous, ≥99%), hexafluorobenzene (HFB, 99%), and 2,2,3,3,4,4,5,5-octafluorohexane-1,6-diol (98%) were obtained from Sigma-Aldrich (Merck KGaA, Darmstadt, Germany). 2,2,3,3,4,4,5,5-Octafluorohexane-1,6-diyl diacrylate (HDDA-F) was purchased from WITHCHEM Co., Ltd. (Seoul, Republic of Korea). Diglycerol diacrylate (DGDA) and isophorone diisocyanate (IPDI) were supplied by URAY Co., Ltd. (Hwaseong-si, Republic of Kora). The photoinitiator, 1-hydroxycyclohexyl phenyl ketone (Irgacure 184), was used for UV curing. All chemicals and solvents were used as received unless otherwise stated.

### 2.2. Synthesis of Mono-Substituted Fluorinated Alcohol (FA)

The mono-substituted fluorinated alcohol (FA) was synthesized by the nucleophilic aromatic substitution (SNAr) of hexafluorobenzene with 2,2,3,3,4,4,5,5-octafluorohexane-1,6-diol, as shown in Scheme S1a (see the Supplementary Materials for details). The reaction was carried out in a 100 mL three-neck round-bottom flask equipped with a dropping funnel, a nitrogen inlet, a water condenser, and a thermometer. HFB (14.2 g, 76.3 mmol, 2.0 equiv), K_2_CO_3_ (6.86 g, 49.6 mmol, 1.3 equiv), and octafluorohexane-1,6-diol (10.0 g, 38.2 mmol, 1.0 equiv) were reacted in anhydrous DMAc at 60 °C for 5 h under a nitrogen atmosphere. An excess of HFB was employed to suppress the di-substitution of both hydroxyl groups of the diol. After the reaction, 3% aqueous HCl was added dropwise with vigorous stirring until the mixture became acidic (pH < 2). The mixture was transferred to a separatory funnel and washed four times with distilled water until the aqueous layer was neutralized, and the organic phase was concentrated under reduced pressure. Because the crude product contained a small amount of the di-substituted byproduct formed by the reaction of both hydroxyl groups with HFB, it was purified by silica gel flash column chromatography using *n*-hexane/ethyl acetate (4:1, *v*/*v*) as the eluent to afford the desired mono-substituted FA (1) as a colorless oil (6.5 g, 40% yield).

### 2.3. Synthesis of Fluorinated Epoxy Acrylate (FEA) Oligomer

**Figure 2:**
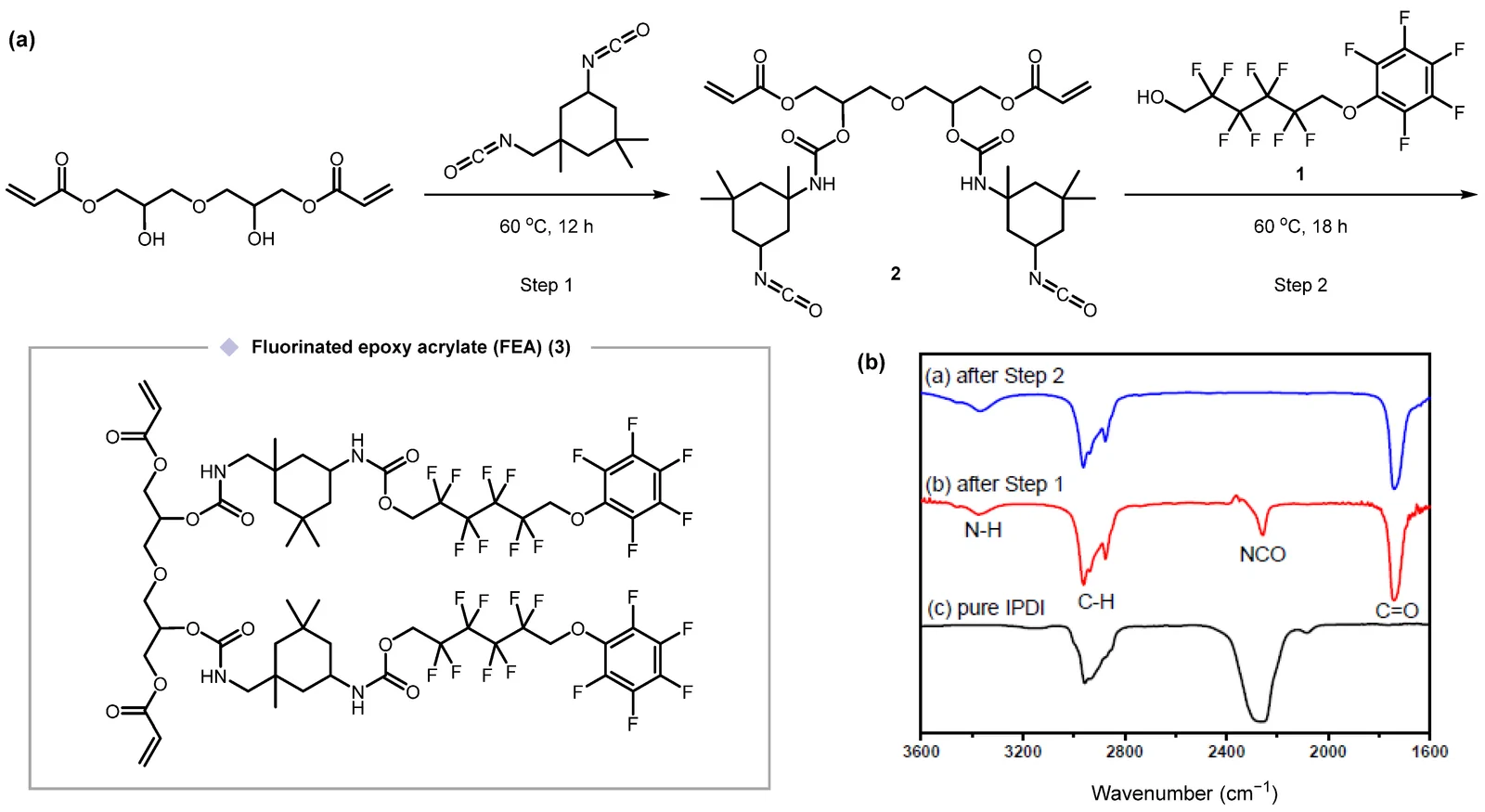
Synthesis and FT-IR characterization of comb-like FEA oligomer. (**a**) Synthetic route and proposed molecular structure of FEA. (**b**) FT-IR spectra of pure IPDI, the Step 1 intermediate, and the final FEA product after Step 2.

The FEA oligomer was synthesized through a two-step urethane-forming reaction between isocyanate (–N=C=O) and hydroxyl (–OH) groups, as shown in Figure 2. In the first step, DGDA (1.37 g, 5.0 mmol, 1.0 equiv) was dissolved in butyl acetate in a 100 mL three-neck round-bottom flask equipped with a reflux condenser, a dropping funnel, and a thermometer under a dry nitrogen purge, and IPDI (2.23 g, 10.0 mmol, 2.0 equiv) was then added slowly with vigorous stirring. Since thermal curing of the acrylate groups occurred above 65 °C, the reaction temperature was maintained at 60 °C for 12 h to form an isocyanate-functional acrylate intermediate (**2**). In the second step, the as-synthesized FA (**1**) (4.29 g, 10.0 mmol, 2.0 equiv) was added dropwise to the reaction mixture under nitrogen purging, and the reaction was continued at 60 °C for 18 h until the NCO stretching peak at approximately 2265 cm^−1^ completely disappeared in the FT-IR spectrum. After completion, IPA was added to quench any residual isocyanate groups, and the solvent and excess IPA were removed under reduced pressure. The final FEA oligomer (**3**) was obtained as a transparent, viscous product (4.26 g, 54% yield).

### 2.4. Preparation of UV-Cured FEA Films

UV-curable formulations were prepared by mixing the synthesized FEA oligomer, HDDA-F as a reactive diluent, and 5 wt% Irgacure 184. The FEA/HDDA-F weight ratios were adjusted to prepare FEA 100, FEA 70, FEA 50, FEA 30, and FEA 0 films; the sample designations, compositions, and theoretical fluorine contents calculated from the chemical structures of the components are summarized in Table S1 (see the Supplementary Materials for details). Each formulation was stirred overnight at room temperature to obtain a homogeneous mixture. A drop of the mixture was then placed between two glass plates separated by a 1 mm thick spacer and irradiated for 30 min in a UV curing chamber (UVACUBE 400, Dr. Hönle AG, Gräfelfing, Germany) under a dry Ar atmosphere to minimize oxygen inhibition, yielding films of approximately 20 × 20 mm^2^ with a thickness of 1 mm. The cured films were kept at room temperature for 10 h before characterization.

### 2.5. Characterization

The chemical structures of the synthesized FA, FEA oligomer, and the resulting UV-cured films were verified via Fourier transform infrared (FT-IR) spectroscopy (Scimitar Series FTS800, Varian Instruments, Randolph, MA, USA). The spectra were recorded using 32 scans over a wavenumber range of 380 to 4000 cm^−1^ with a spectral resolution of 4 cm^−1^. Additionally, proton (^1^H) and fluorine (^19^F) nuclear magnetic resonance (NMR) spectra were acquired on a 300 MHz spectrometer (Bruker Avance 300 MHz spectrometer, Bruker, Billerica, MA, USA) using CDCl_3_ as the deuterated solvent.

Surface wettability and free energy were assessed by measuring the contact angles of water and ethylene glycol at room temperature using a CAM100 optical contact angle meter (KSV Instruments Ltd., Helsinki, Finland). To ensure reliability, measurements were repeated a minimum of three times at different locations on each film, carefully avoiding droplet distortion or environmental vibration. The total surface free energy was determined using the geometric-mean approach proposed by Owens and Wendt [24]. The mathematical relationship is expressed as Equation (1):

(1) $$\left(1+\cos\theta \right)\gamma_{l}={2\left(\gamma_{s}^{d}\gamma_{l}^{d}\right)}^{1/2}+\;{2\left(\gamma_{s}^{nd}\gamma_{l}^{nd}\right)}^{1/2}$$

where γ_s_ and γ_l_ denote the surface free energies of the solid surface and the testing liquid, respectively. The superscripts ‘d’ and ‘nd’ represent the dispersive and non-dispersive (polar) components. The effective contact angle (θ) utilized in this model was derived from the advancing and receding angles using Equation (2):

(2) $$\theta ={cos}^{-1}\left(\frac{{cos\theta }_{a}+\;{cos\theta }_{r}}{2}\right)$$

For the calculations, the reference energy values for water were set to γ_l_ = 72.8 mJ/m^2^, γ_l_^d^ = 21.8 mJ/m^2^, and γ_l_^nd^ = 51.0 mJ/m^2^. For ethylene glycol, the constants used were γ_l_ = 48.0 mJ/m^2^, γ_l_^d^ = 29.0 mJ/m^2^, and γ_l_^nd^ = 19.0 mJ/m^2^.

The microscopic surface features of the films were examined using a field emission scanning electron microscope (FE-SEM; JSM-6700F, JEOL Ltd., Tokyo, Japan). All specimens were coated with platinum (Pt) prior to imaging to prevent thermal degradation and melting caused by the electron beam.

The surface chemical composition of the UV-cured FEA 100 film was investigated by X-ray photoelectron spectroscopy (XPS; NEXSA G2, Thermo Fisher Scientific Waltham, MA, USA) using a monochromatic Al Kα source (1486.6 eV). Spectra were collected from the air-facing side of the film. Peak fitting was performed following Shirley background subtraction with constrained peak shapes and full widths at half maximum, and binding energies were referenced to the C–C/C–H component at 284.3 eV.

Thermal degradation behaviors were monitored using a thermogravimetric analyzer (Q600, TA Instruments, New Castle, DE, USA). The samples were heated from room temperature up to 550 °C at a constant ramp rate of 10 °C/min under a continuous nitrogen flow of 100 mL/min.

Finally, the photopolymerization kinetics were evaluated via photo-differential scanning calorimetry (photo-DSC). The curing process was conducted at room temperature under a UV intensity of 10 mW/cm^2^ for a duration of 3 min. To eliminate the oxygen inhibition effect typical of acrylate radicals, the DSC chamber was continuously purged with nitrogen gas at a flow rate of 50 mL/min during the measurement. After the first irradiation, the sample was cooled to room temperature and re-irradiated under identical conditions, and the enthalpy evolved in this second run was taken as the residual heat used in Equation (3).

## 3. Results and Discussion

### 3.1. Synthesis and Structural Characterization of FEA

The comb-like fluorinated epoxy acrylate (FEA) oligomer was synthesized through a two-step urethane-forming reaction, as shown in Figure 2. In the first step, diglycerol diacrylate (DGDA) was reacted with isophorone diisocyanate (IPDI) to form an isocyanate-functional acrylate intermediate. In the second step, the remaining isocyanate groups were reacted with the mono-substituted fluorinated alcohol (FA), resulting in the incorporation of fluorinated side chains terminated with perfluorophenyl groups onto the acrylate backbone. The final FEA oligomer thus combines UV-curable acrylate end groups, urethane linkages, and perfluorinated side chains bearing aromatic perfluorophenyl termini in a comb-like molecular architecture.

Recent synthetic approaches include solvent-free ring opening/annulation reactions involving P(O)–OH compounds and epichlorohydrocarbons [25], as well as cyclic carbonate-amine chemistry for preparing non-isocyanate polyurethane foams from solvent-free formulations [26]. In comparison, the present IPDI–alcohol route enables stepwise incorporation of the fluorinated side chains while preserving acrylate groups for subsequent UV curing. However, oligomer synthesis still uses isocyanates and organic solvents; solvent-free processing is limited to the subsequent formulation and curing stages, and the principal contribution is fluorine-efficient molecular design rather than a fully solvent-free or non-isocyanate synthesis.

FT-IR spectroscopy was used to monitor the reaction progress and confirm the formation of the FEA oligomer (Figure 2b). Pure IPDI showed a strong isocyanate stretching band at approximately 2265 cm^−1^. After Step 1, the intensity of this NCO peak decreased, indicating partial consumption of the isocyanate groups by reaction with the hydroxyl groups of DGDA. After Step 2, the NCO peak completely disappeared, confirming that the remaining isocyanate groups had reacted with FA. In addition, the appearance of N–H (~3350 cm^−1^) and urethane C=O (~1700–1750 cm^−1^) stretching bands supported the formation of urethane linkages. The weak, broad absorption in the O–H stretching region observed in the final spectrum is attributed to atmospheric moisture adsorbed during the measurement rather than to unreacted hydroxyl groups, as the complete disappearance of the NCO band confirms full conversion of the isocyanate functionality.

The chemical structures of FA and the FEA oligomer were further confirmed by ^1^H and ^19^F NMR spectroscopy (Figures S1–S4, Supplementary Materials). In the ^1^H NMR spectrum of the FEA oligomer, the characteristic vinyl protons of the acrylate groups were observed at δ = 6.40, 6.10, and 5.84 ppm, and the urethane N–H protons appeared in the δ = 5.35–4.77 ppm region, while the methylene protons adjacent to the ether and carbamate linkages were detected at δ = 4.57–3.50 ppm. In the ^19^F NMR spectrum, the multiplets at δ = −120.1 to −124.7 ppm were assigned to the –CF_2_– units of the octafluorohexylene segments, and the resonances at δ = −156.4, −162.2 to −163.2, and −160.9 to −161.8 ppm corresponded to the ortho-, meta-, and para-fluorine atoms of the pentafluorophenoxy end groups, respectively. The integration ratios, consistent with the proposed structure, further verified the successful synthesis of the mono-substituted FA and the target comb-like FEA oligomer.

These results collectively demonstrate the successful synthesis of the proposed FEA oligomer. The acrylate end groups are expected to participate in UV-induced crosslinking, while the fluorinated side chains can preferentially migrate toward the air–polymer interface during film formation and curing owing to their low surface energy [14,15,16]. Therefore, the comb-like FEA structure was designed to provide both efficient UV curability and a low-surface-energy film surface.

### 3.2. FT-IR Analysis of UV-Cured FEA/HDDA-F Films

**Figure 3:**
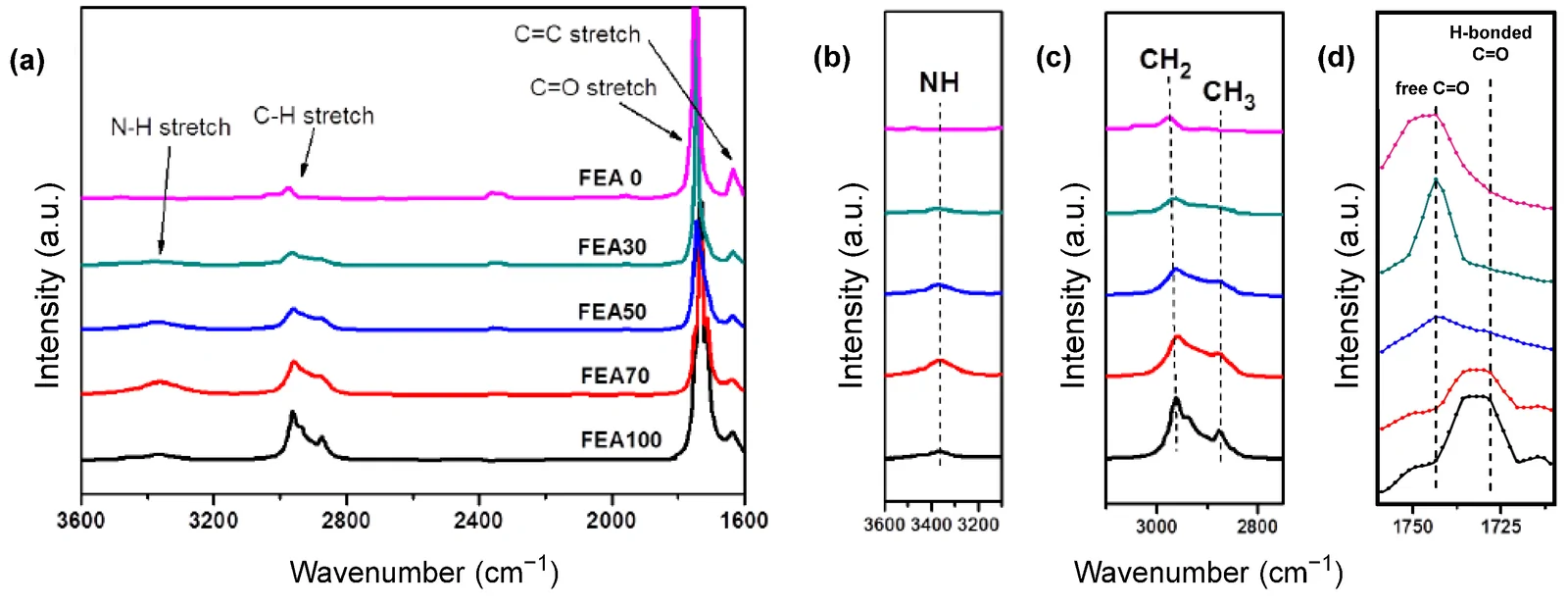
FT-IR spectra of UV-cured FEA/HDDA-F films. (**a**) Full spectra, (**b**) N–H stretching region, (**c**) C–H stretching region, and (**d**) C=O stretching region.

The chemical structures of the UV-cured FEA/HDDA-F films were further investigated by FT-IR spectroscopy, as shown in Figure 3. All cured films exhibited characteristic absorption bands corresponding to the acrylate-based fluorinated network. The broad N–H stretching band appeared in the range of 3250–3500 cm^−1^, indicating the presence of urethane linkages in the FEA-containing films. The C–H stretching bands of CH_2_ and CH_3_ groups were observed at approximately 2960 and 2870 cm^−1^, respectively. In addition, the C=C stretching band of the acrylate group and the C=O stretching band were observed near 1635 and 1728–1745 cm^−1^, respectively. Notably, the strong absorptions in the region of 1100–1250 cm^−1^, assigned to the C–F stretching vibrations of the –CF_2_– segments and the perfluorophenyl rings [11], were present in all compositions, providing direct evidence of the fluorinated moieties in the cured networks.

The enlarged spectra provide more detailed information on the effect of FEA content. As shown in Figure 3b, the N–H stretching band became more evident with increasing FEA content, which is attributed to the urethane groups introduced through the DGDA–IPDI–FA reaction sequence. In the C–H stretching region (Figure 3c), the CH_2_ and CH_3_ signals also became more pronounced in FEA-rich films because of the IPDI-derived aliphatic segments. In the carbonyl region (Figure 3d), the relative intensity of the hydrogen-bonded C=O band at 1728 cm^−1^ increased as the FEA content increased, whereas the free C=O contribution at 1745 cm^−1^ became more dominant in HDDA-F-rich compositions. This result suggests that the urethane groups in the FEA oligomer promote intermolecular interactions through hydrogen bonding within the cured network.

Overall, the FT-IR spectra confirm the successful formation of UV-cured FEA/HDDA-F films and demonstrate that the chemical environment of the cured network changes systematically with the FEA content. The increased urethane-related absorption bands in FEA-rich films support the incorporation of the comb-like FEA oligomer into the crosslinked coating structure.

### 3.3. Photopolymerization Behavior of the FEA/HDDA-F Formulations

**Figure 4:**
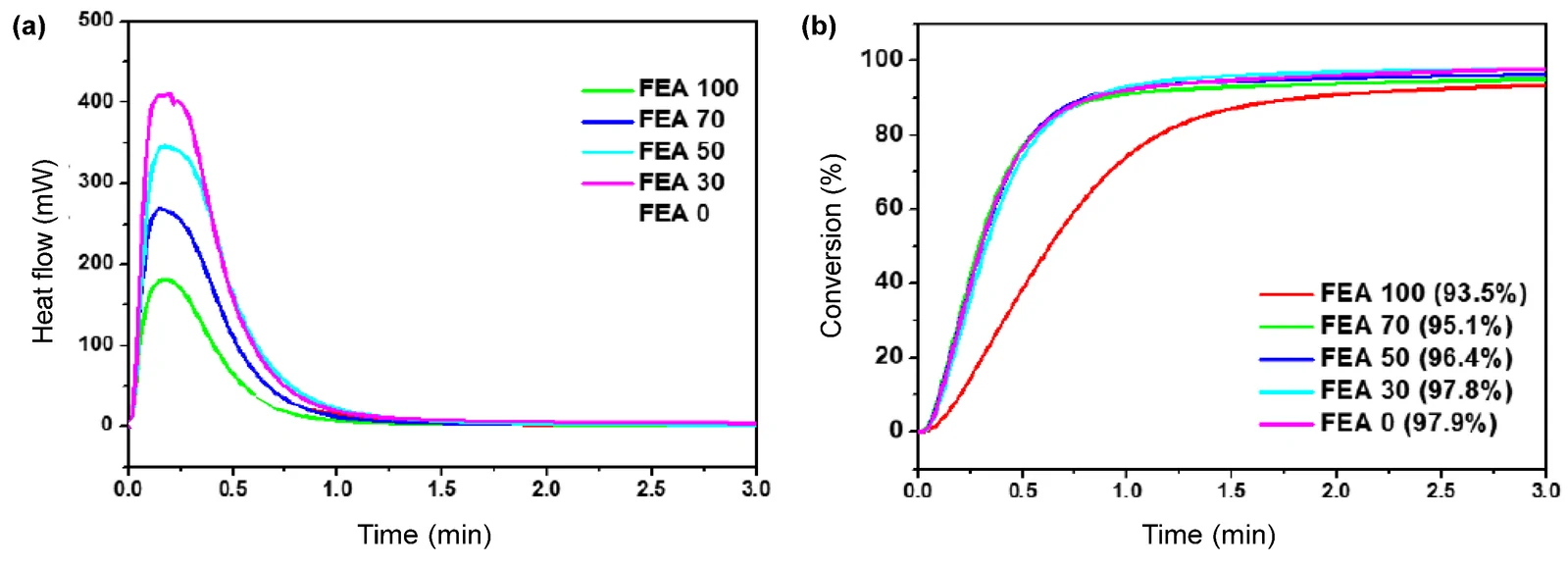
Photopolymerization behavior of UV-curable FEA/HDDA-F formulations. (**a**) Heat flow profiles and (**b**) conversion curves obtained by photo-DSC under UV irradiation.

To monitor the photopolymerization kinetics and the degree of curing of the formulations, photo-differential scanning calorimetry (photo-DSC) was conducted, and the resulting heat flow profiles and conversion curves are presented in Figure 4.

Figure 4a displays the heat flow profiles recorded during UV irradiation. All formulations exhibited a rapid exothermic response upon UV irradiation, with the maximum heat flow reached within 0.5 min, confirming the fast curing characteristic of the acrylate-based system. The peak intensity of the exothermic heat flow decreased as the proportion of the FEA oligomer in the formulation increased. While this reduction might initially appear to imply poor UV-curing characteristics for the newly synthesized FEA, it is fundamentally attributed to the structural and compositional differences between the formulation components. Specifically, since the molecular weight of the FEA oligomer (MW = 1575.13 g/mol) is approximately 4.25 times higher than that of the reactive diluent HDDA-F (MW = 370.19 g/mol), a higher FEA content results in a considerably lower concentration of polymerizable acrylate double bonds per unit weight of the sample. This reduced double-bond density directly leads to the diminished intensity of the heat flow during the polymerization process.

The final degree of conversion (%) for each formulation was calculated from the photo-DSC heat flow data using Equation (3):

(3) $$Conversion\left(\%\right)=\frac{{∆H}_{total}-{∆H}_{residue}}{{∆H}_{total}}\times 100$$

where ΔH_total_ is the sum of the enthalpies evolved in the first and second irradiation runs, and ΔH_residue_ is the enthalpy evolved in the second run from acrylate groups left unreacted after the initial cure.

As plotted in the conversion curves (Figure 4b), all formulations exhibited highly efficient photopolymerization despite the reduced heat flow intensity. The ultimate degrees of conversion were 97.9%, 97.8%, 96.4%, and 95.1% for FEA 0, FEA 30, FEA 50, and FEA 70, respectively, and even the FEA 100 sample, which consists entirely of the synthesized oligomer without any reactive diluent, reached a highly respectable curing degree of 93.5%. The slightly lower conversion of FEA 100 is attributed to the increased viscosity and the steric bulkiness of the oligomer, which restrict the mobility of the propagating radicals in the later stage of polymerization [27]. Nevertheless, a conversion exceeding 93% for the neat FEA oligomer demonstrates that the newly synthesized comb-like FEA retains excellent UV curability, validating its use as a primary oligomer in UV-curable coating formulations.

### 3.4. Thermal Stability and Degradation Behavior of UV-Cured FEA Films

**Figure 5:**
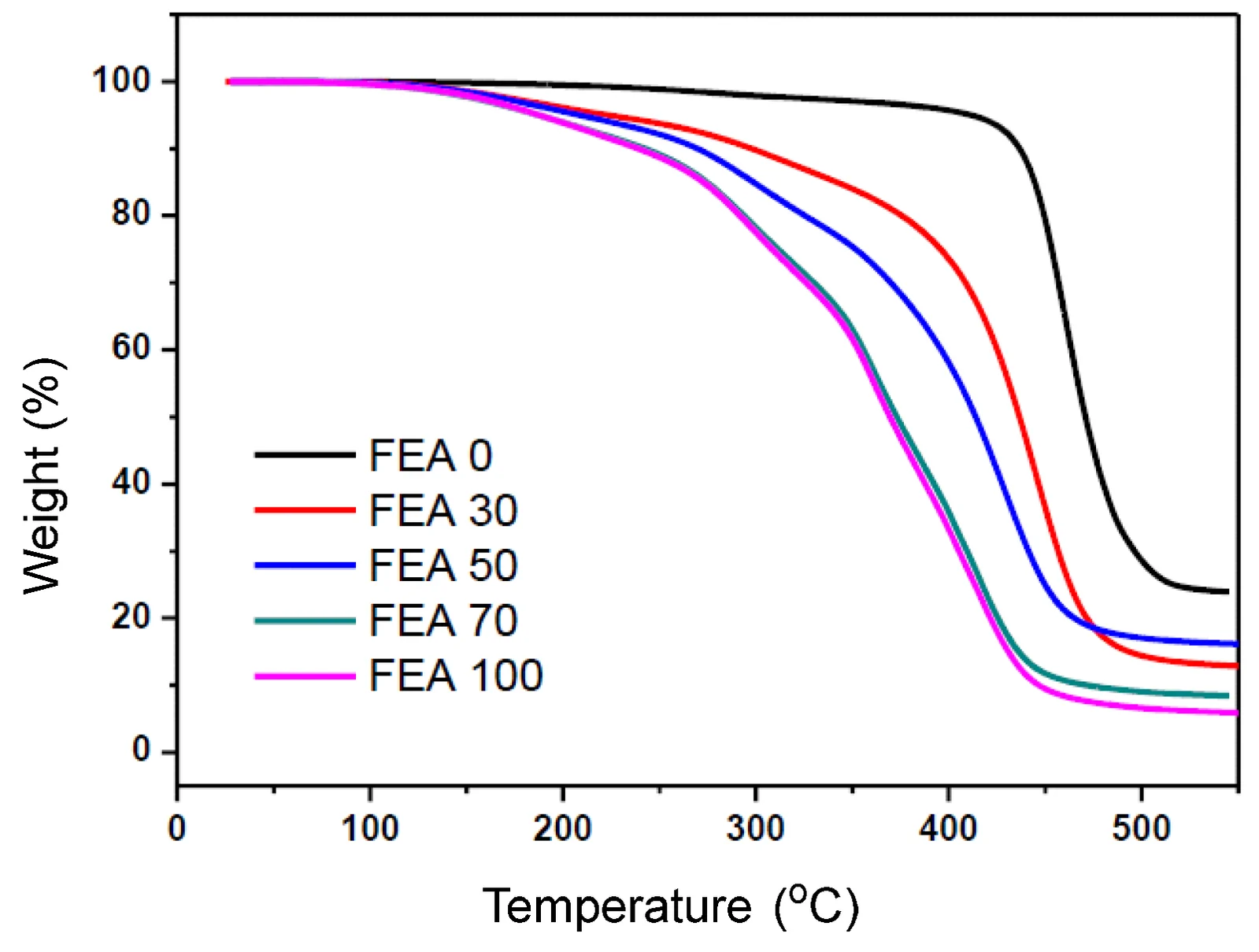
Thermal stability of UV-cured FEA films.

To evaluate the thermal stability of the UV-cured films, thermogravimetric analysis (TGA) was conducted from room temperature to 550 °C, and the corresponding thermograms are presented in Figure 5. The characteristic thermal degradation parameters, including the temperature at 5% weight loss (T_d5%) and the maximum degradation temperatures (T_max1 and T_max2), were determined from the thermograms.

The TGA curves exhibit two distinct degradation regions, indicating a two-step thermal degradation process. The first degradation step, occurring at approximately 250–350 °C, is assigned to the cleavage of the thermally labile urethane linkages; the dissociation of the N–C(O) bond of the carbamate group is known to initiate at relatively low temperatures (typically 200–300 °C) [28,29]. The second step at 380–470 °C corresponds to the disintegration and breakdown of the crosslinked acrylate backbone.

Comparing the thermal behavior according to the film composition, a clear trend is observed: T_d5% decreased from 390 °C for FEA 0 to 260 °C for FEA 100 as the proportion of the FEA oligomer increased. This trend is primarily attributed to the increasing density of urethane linkages in FEA-rich films, since the FEA oligomer introduces four carbamate groups per molecule, whereas HDDA-F contains none. FEA 70 and FEA 100 displayed nearly identical TGA profiles, suggesting that the urethane content approaches saturation in its effect on the initial degradation behavior at high FEA loadings. It is worth noting that all films remained essentially stable (weight loss < 5%) up to approximately 250 °C, which is well above the service temperature range of typical protective and decorative coatings. Therefore, although the incorporation of the urethane-rich FEA oligomer slightly lowers the onset of degradation relative to the pure HDDA-F network, the thermal stability of the FEA films remains fully adequate for practical coating applications.

### 3.5. Optical Appearance and UV-Induced Yellowing Behavior

**Figure 6:**
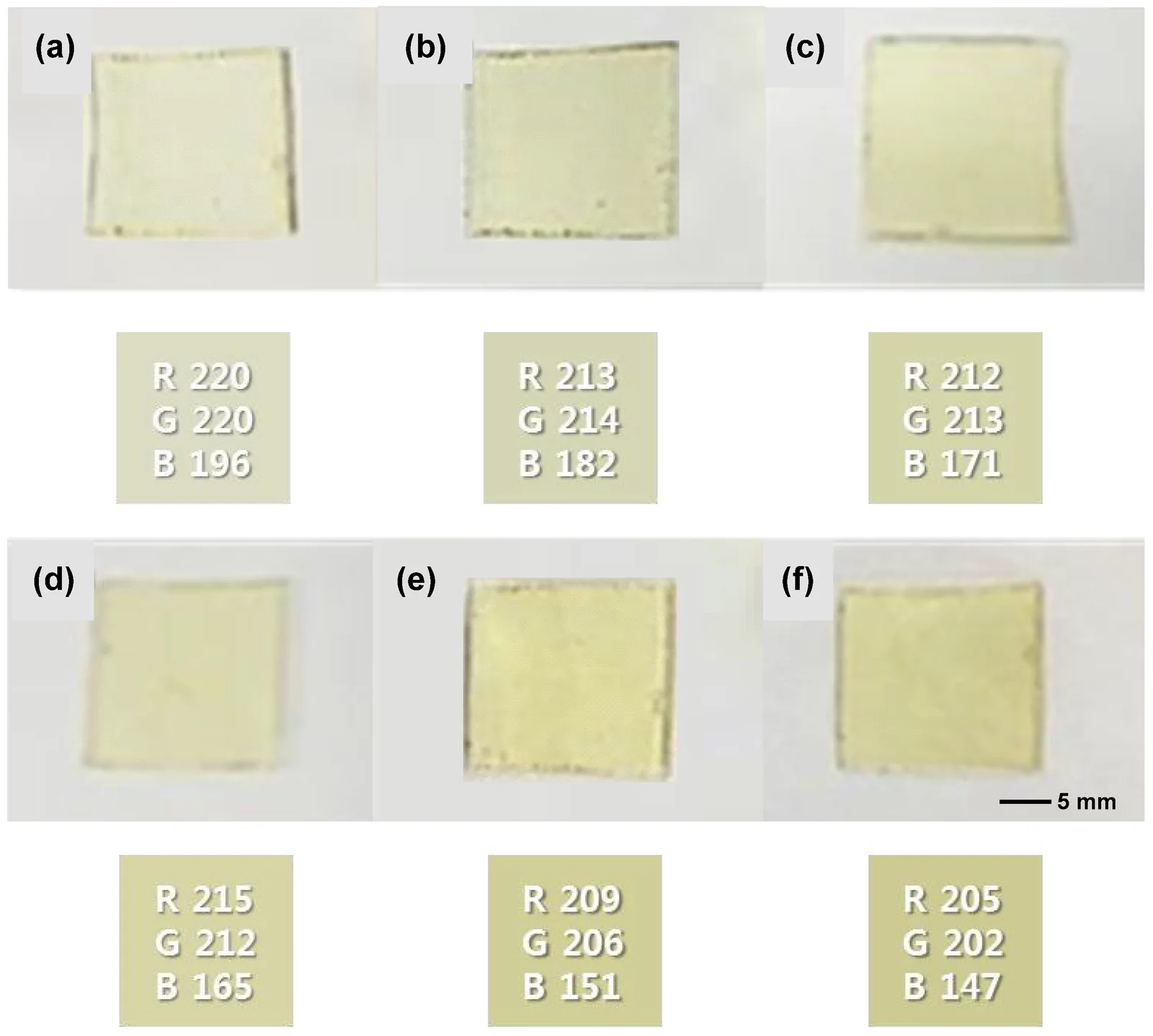
Optical appearance and yellowing behavior of FEA 100 film under prolonged UV irradiation. Photographs and RGB values of the FEA 100 film specimen after UV irradiation for (**a**) 0.5 h, (**b**) 1 h, (**c**) 1.5 h, (**d**) 2 h, (**e**) 2.5 h, and (**f**) 3 h. Scale bar: 5 mm (panel (**f**)).

The UV resistance and changes in the optical appearance of the FEA 100 films under prolonged UV irradiation were investigated. Figure 6 presents photographs of the films continuously exposed to UV light for intervals ranging from 0.5 to 3 h, alongside the RGB values extracted from each state. All photographs were taken under identical illumination and camera conditions to allow relative comparison of the color coordinates.

As visually apparent, a gradual yellowing phenomenon was observed in the films as the UV irradiation time increased. This visual color change is consistent with the variations in the extracted RGB values: with longer exposure times, the B (blue) channel value in particular decreased markedly from 196 after 0.5 h to 147 after 3 h, indicating increased absorption in the blue region of the visible spectrum, which is perceived as yellowing. The photo-yellowing of UV-cured acrylate systems is generally ascribed to the photolysis products of the residual photoinitiator and the photo-oxidation of urethane segments, which generate chromophoric species such as quinoid structures upon prolonged irradiation [30,31].

Although gradual yellowing occurred, no visible cracks or delamination were observed after 3 h of UV irradiation (Figure 6f). This observation indicates short-term visual integrity under the tested conditions, rather than established long-term UV durability. For comparison, recent CeO_2_@GO-modified epoxy coatings were evaluated using 168 h UV-aging tests and 60-day saline immersion tests, with electrochemical measurements used to assess corrosion protection [32]. Accelerated weathering, chemical exposure, and cyclic wetting–drying tests remain necessary to establish the service durability of the present FEA films.

### 3.6. Surface Wettability, Surface Free Energy, and Morphology

**Figure 7:**
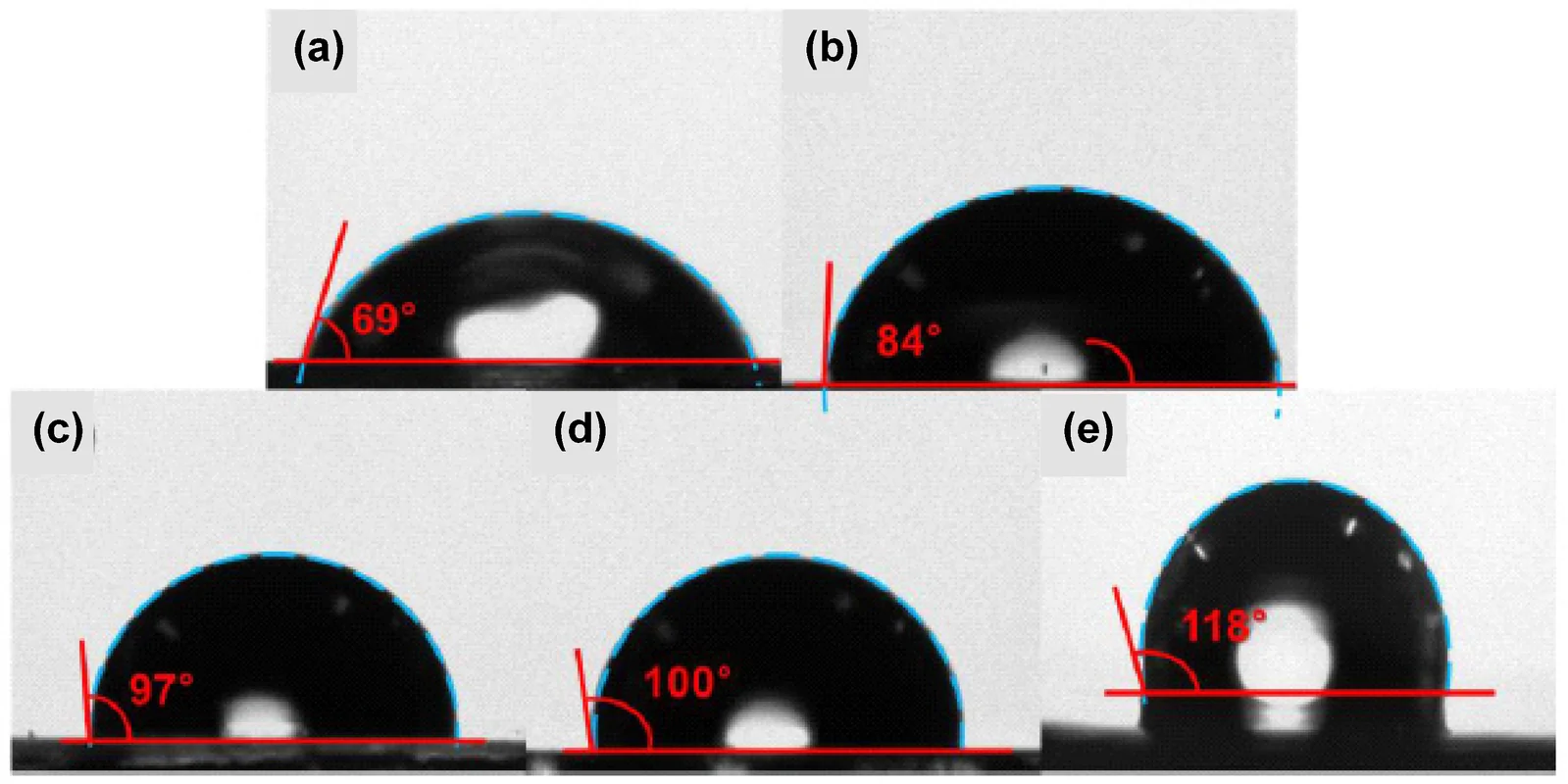
Surface wettability of UV-cured FEA films. Advancing water contact angle images of FEA films with different FEA contents: (**a**) FEA 0, (**b**) FEA 30, (**c**) FEA 50, (**d**) FEA 70, and (**e**) FEA 100. The red lines and values indicate the measured advancing water contact angles.

To evaluate the hydrophobicity of the cured films, the advancing water contact angles (θ_a_) were first measured. As shown in Figure 7, θ_a_ increased dramatically from 69° for FEA 0 to 118° for FEA 100 as the FEA content increased. Given that a contact angle above 90° indicates a hydrophobic surface, it is noteworthy that the advancing contact angle already exceeded this threshold at the FEA 50 composition (97°), and for the FEA 100 film even the receding angle (105°) remained well above 90°, while FEA 30 (84°) also showed a substantial improvement over the fluorinated diluent alone. To quantify the surface free energy via the geometric-mean approach described in Section 2.5, the corresponding receding (θ_r_) contact angles of water and the advancing and receding contact angles of ethylene glycol were also measured, and the results are summarized in Table 1.

The calculated total surface free energy decreased markedly from 34.0 mJ/m^2^ for FEA 0 to 24.5 mJ/m^2^ upon the incorporation of only 30 wt% of the FEA oligomer, remained nearly constant at the intermediate compositions (23.7 and 24.4 mJ/m^2^ for FEA 50 and FEA 70, respectively), and reached a minimum of 19.2 mJ/m^2^ for the FEA 100 film, which is comparable to that of polytetrafluoroethylene (PTFE, 18.5 mJ/m^2^) [33]. It should be emphasized that this superior surface performance was achieved despite a lower overall fluorine content: as shown in Table S1, the total fluorine content of the FEA 100 formulation (31.4 wt%) is in fact lower than that of the FEA 0 formulation (41.1 wt%). In other words, the comb-like FEA oligomer delivers markedly higher hydrophobicity and lower surface energy per unit of incorporated fluorine than the conventional fluorinated diluent, directly demonstrating the enhanced fluorine efficiency targeted by the present molecular design.

Table 2 compares the present system with the fluorinated poly(urethane-acrylate)s of Lin et al. [10], which were evaluated using the same Owens–Wendt treatment with water and ethylene glycol as test liquids. At an essentially identical fluorine loading (32.8 wt% for their H-100 versus 31.4 wt% for FEA 100), the backbone-fluorinated system reaches an advancing water contact angle of 95.2°, whereas the comb-like FEA attains 118°. Their M-100 film shows a comparable total surface free energy (19.1 mJ/m^2^), but this arises from a combination of dispersive (8.4 mJ/m^2^) and polar (10.7 mJ/m^2^) contributions, whereas the polar component of FEA 100 is only (0.2 mJ/m^2^). The improvement in water repellency therefore arises from the placement of the fluorinated segments rather than from a higher fluorine content.

**Figure 8:**
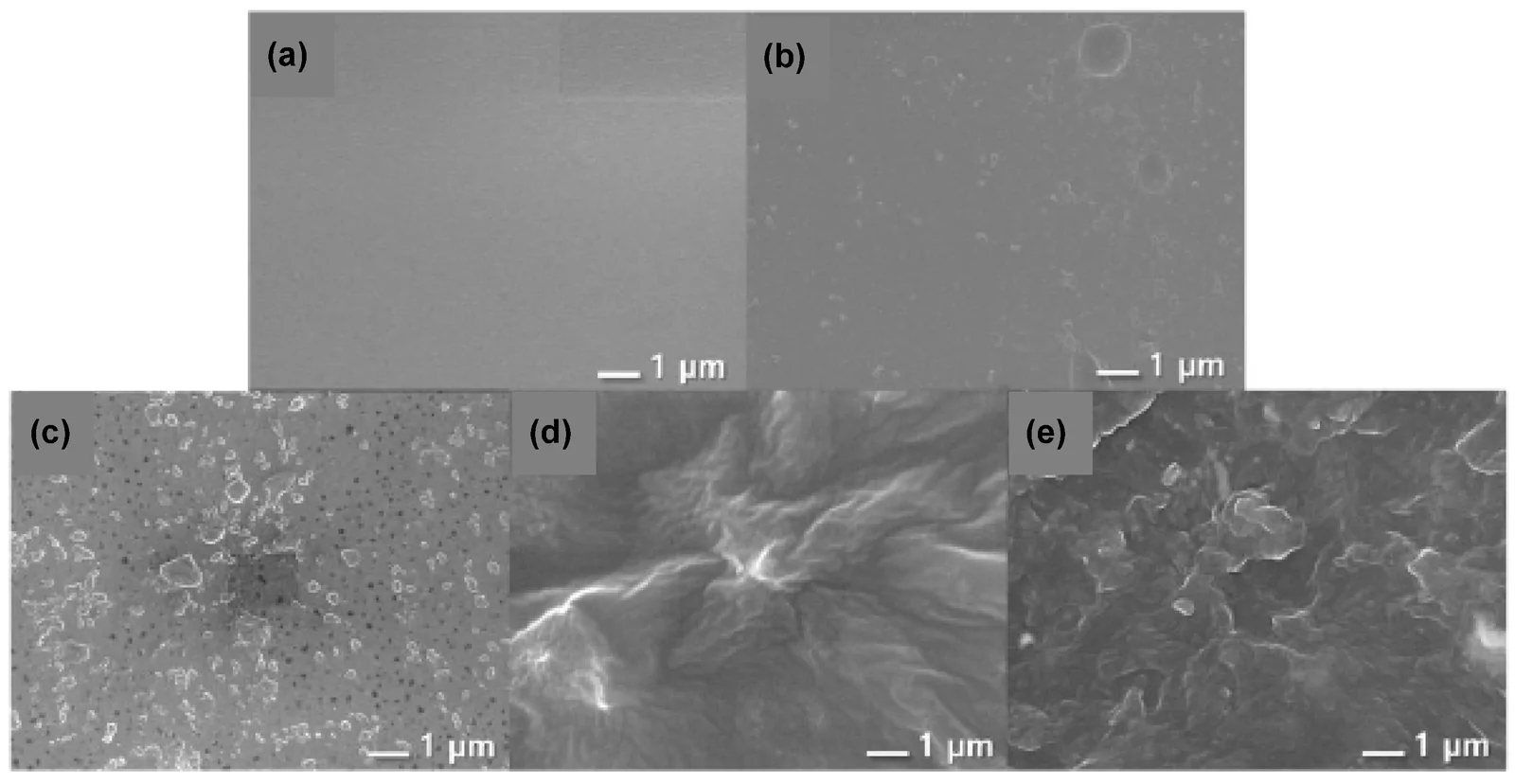
Surface morphology of UV-cured FEA films. FE-SEM images of FEA films with different FEA contents: (**a**) FEA 0, (**b**) FEA 30, (**c**) FEA 50, (**d**) FEA 70, and (**e**) FEA 100.

Further insight into the surface chemistry is provided by the decomposition of the surface free energy into its dispersive (γ_s_^d^) and non-dispersive (γ_s_^nd^) components, which exhibit markedly different composition dependences. The non-dispersive (polar) component decreased monotonically and drastically from 23.9 mJ/m^2^ for FEA 0 to nearly zero (0.2 mJ/m^2^) for FEA 100, and this suppression accounts for essentially all of the overall reduction in the total surface free energy. In contrast, the dispersive component showed no systematic trend with composition, varying between 9.0 and 21.8 mJ/m^2^; the apparent increase for the FEA 50–100 films is likely associated with the micro-scale surface roughness developed in these compositions (Figure 8), which affects the apparent contact angles used in the Owens–Wendt analysis, rather than with a fundamental change in the dispersive surface chemistry. Since polar contributions arise from surface functional groups such as carbonyl, ether, and urethane moieties, the near-complete and monotonic suppression of γ_s_^nd^ indicates that these polar groups are effectively screened from the outermost surface, which is consistent with the preferential enrichment of the nonpolar perfluorinated side chains at the air–film interface [14,15,16]. The contact angle hysteresis (θ_a_–θ_r_) of water remained moderate, in the range of 6–13° for the FEA-containing films, suggesting that the outermost surface combines a fluorine-enriched chemistry with the micro-scale roughness developed during curing; the slight increase in hysteresis for the FEA 70 and FEA 100 films (10–13°) is consistent with the progressively rougher, wrinkled morphologies observed by FE-SEM (Figure 8), as surface texturing is known to enhance contact line pinning.

**Figure 9:**
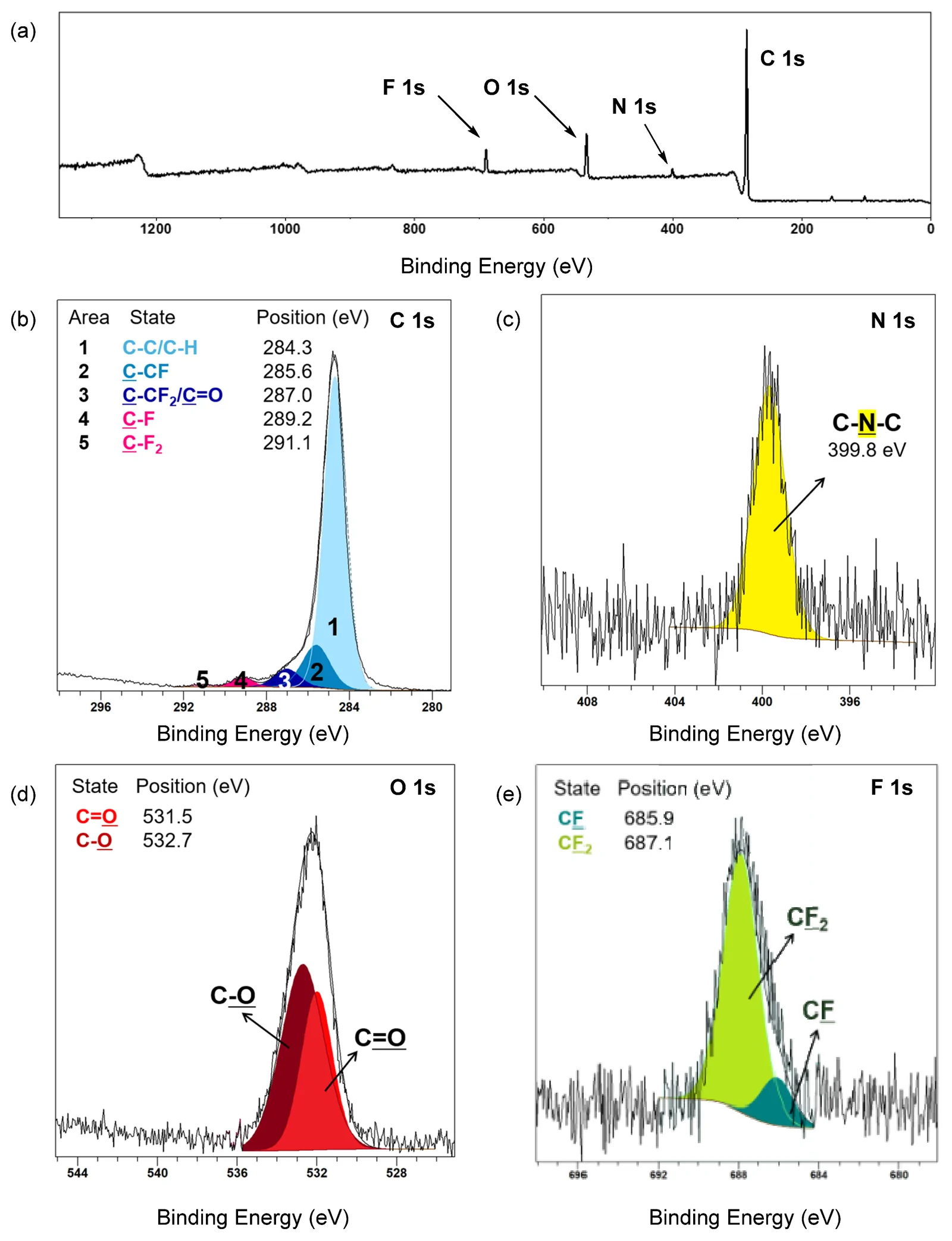
XPS spectra of the UV-cured FEA 100 film: (**a**) survey spectrum and high-resolution deconvoluted spectra of (**b**) C 1s, (**c**) N 1s, (**d**) O 1s, and (**e**) F 1s, showing the chemical states of the corresponding elements.

To examine the surface fluorine enrichment and side chain segregation, high resolution X-ray photoelectron spectroscopy (XPS) analysis was performed on the cured FEA 100 film as shown in Figure 9. The high-resolution C 1s spectrum is clearly deconvoluted into five chemical states, establishing a direct correlation with the molecular structure of fluorinated epoxy acrylate in Figure 2. The sharp peaks at 291.1 eV and 289.2 eV correspond to the C–F_2_ groups of the aliphatic octafluorohexylene spacers and the C–F bonds of the terminal perfluorophenyl rings, respectively. Additionally, the peaks at 287.0 eV(C–CF_2_/C=O), 285.6 eV(C–CF), 284.3 eV(C–C/C–H) trace the core carbon skeleton of the crosslinked network. The F 1s spectrum exhibits two components at 687.1 eV (CF_2_) and 685.9 eV (CF), consistent with the presence of both aliphatic and aromatic fluorinated moieties in the near-surface region. The presence of the C–N–C carbamate linkage at 399.8 eV in the N 1s spectrum, alongside the ester/urethane oxygen states in the O 1s spectrum in 531.5 eV for C=O and 532.7 eV for C–O, confirm that the comb-like FEA oligomer is successfully interconnected within the matrix. These XPS results confirm the presence of fluorinated moieties in the near-surface region of the cured FEA 100 film. Together with the wettability and FE-SEM results, they support the proposed preferential surface segregation of the fluorinated side chains. Such segregation may reduce the exposure of polar urethane and carbonyl groups at the film surface, contributing to the observed hydrophobicity. It should be noted, however, that the present analysis was performed on the air-facing side of the FEA 100 film only; depth-resolved techniques such as angle-resolved XPS or ToF-SIMS would be required to quantify the extent of enrichment. This selective segregation effectively screens the polar urethane and carbonyl groups from the surface, leading to the hydrophobicity on the cured film.

The surface morphology of the films was then examined by FE-SEM to correlate the wetting behavior with the surface topography (Figure 8). The FEA 0 film exhibited a featureless, smooth surface, consistent with the absence of fluorinated side chains in the pure HDDA-F network. In contrast, with increasing FEA content, the films developed progressively rougher, lumpy, and textured surfaces, and the FEA 100 film—consisting entirely of the synthesized oligomer—displayed a distinctly wrinkled and bumpy morphology. This morphological transformation is likely attributed to the self-organization of the comb-like oligomer during UV curing: as the acrylate backbones are crosslinked, the perfluorinated side chains, which are immiscible with the polymerizing acrylate matrix, segregate toward the free surface, generating micro-scale surface irregularities [14,15,16].

Based on these observations, the enhanced hydrophobicity of the FEA-rich films can be rationalized by two cooperative factors. The primary factor is the chemical enrichment of the low-surface-energy perfluorinated side chains at the outermost surface, as evidenced by the near-zero polar component of the surface free energy. The secondary factor is the surface roughness developed in the FEA-rich films; according to the Wenzel model, roughness amplifies the intrinsic wetting character of a surface, and therefore it further increases the apparent contact angle for the intrinsically hydrophobic (θ > 90°) FEA 50–100 surfaces [34]. Overall, the combination of the systematically suppressed polar surface energy component, the composition-dependent surface texturing, and the fluorine-efficiency comparison supports the interpretation that the comb-like architecture drives the fluorinated side chains toward the film surface, thereby achieving PTFE-like surface properties at a reduced fluorine content.

While the FEA films exhibited excellent static hydrophobicity and low surface free energy, comprehensive dynamic liquid repellency—including sliding angles, droplet mobility, and anti-fouling performance—plays a critical role in advanced self-cleaning applications. Such dynamic droplet behaviors are heavily influenced by the interplay between intrinsic surface chemistry and hierarchical surface roughness [22,35]. Although the detailed investigation of dynamic droplet impact and contamination removal efficiency is beyond the scope of the present synthetic study, it remains a highly promising avenue for future work aimed at optimizing the surface texturing of FEA-based coatings.

Recent UV-cured polyurethane acrylate coatings containing PTFE particles have demonstrated reduced friction and wear through solid-lubricant incorporation [36]. By contrast, the present FEA system targets fluorine-efficient control of wettability through molecular architecture, and the measured contact angles and surface energies should not be interpreted as evidence of low friction or wear resistance. Hardness, scratch and abrasion resistance, adhesion, and frictional behavior therefore remain to be evaluated before practical protective-coating performance can be established.

## 4. Conclusions

In this study, a novel UV-curable fluorinated epoxy acrylate (FEA) oligomer featuring a comb-like architecture with perfluorophenyl-terminated fluorinated side chains was successfully synthesized and evaluated for low-surface-energy coating applications. A mono-substituted fluorinated alcohol was first prepared by the nucleophilic aromatic substitution of hexafluorobenzene with octafluorohexane-1,6-diol, and the FEA oligomer was then constructed through a controlled two-step urethane-forming reaction with diglycerol diacrylate and isophorone diisocyanate. The chemical structure of the oligomer was confirmed by FT-IR and ^1^H/^19^F NMR spectroscopy.

Despite the incorporation of bulky fluorinated side chains, the synthesized FEA retained excellent photopolymerization behavior, achieving a high double-bond conversion of 93.5% for the neat oligomer film, while all FEA/HDDA-F formulations exceeded 93% conversion. The cured films exhibited a two-step thermal degradation governed by the urethane linkages yet remained stable up to approximately 250 °C, which is sufficient for practical coating applications, and they preserved their structural integrity without cracking or delamination even after 3 h of continuous UV irradiation.

Most notably, the cured FEA films demonstrated exceptional surface performance. As the FEA content increased, the advancing water contact angle rose from 69° to 118°, and the total surface free energy decreased from 34.0 to a minimum of 19.2 mJ/m^2^, a value comparable to that of polytetrafluoroethylene (18.5 mJ/m^2^). Importantly, this superior hydrophobicity was achieved at a lower overall fluorine content than that of the fluorinated diluent alone (31.4 vs. 41.1 wt%). The reduction in surface free energy was governed almost entirely by the monotonic, near-complete suppression of its polar component (23.9 → 0.2 mJ/m^2^). The XPS results, together with the wettability data and FE-SEM observations, support the proposed preferential segregation of the fluorinated side chains toward the film surface.

These findings demonstrate that the rationally designed comb-like FEA architecture effectively maximizes fluorine efficiency, delivering PTFE-like surface properties at a reduced fluorine loading, and the synthesized FEA oligomer is therefore a promising candidate for advanced UV-curable hydrophobic, self-cleaning, and water-repellent coatings.

Several aspects nevertheless remain to be addressed. Quantitative topography analysis by atomic force microscopy remains to be performed. Mechanical and tribological properties, including hardness, adhesion, friction, and wear resistance, require further evaluation [36]. Long-term environmental durability, chemical resistance, and corrosion-protection performance should also be assessed [32]. Dynamic wetting behavior, self-cleaning, and multifunctional coating applications remain to be investigated. Photolithographic patterning of thin FEA films and the attainable line width are further directions for future study.

## Figures and Tables

**Table 1:** Contact angles and surface free energies of the UV-cured FEA films.

Material	Water (^o^)		Ethylene Glycol (^o^)		Surface Energy (mJ/m^2^)		
	θ_a_	θ_r_	θ_a_	θ_r_	γ_s_^d^	γ_s_^nd^	γ_s_
FEA 0	69	68	56	50	10.1	23.9	34.0
FEA 30	84	78	74	60	9.0	15.4	24.5
FEA 50	97	90	75	64	20.3	3.4	23.7
FEA 70	100	90	75	65	21.8	2.6	24.4
FEA 100	118	105	95	79	19.0	0.2	19.2

θ_a_ and θ_r_ = advancing and receding contact angle, respectively. γ_s_^d^ and γ_s_^nd^ = dispersive and non-dispersive (polar) component of the surface free energy; γ_s_ = total surface free energy. All values were obtained by the Owens–Wendt geometric-mean method using water and ethylene glycol as test liquids, with the effective contact angle calculated from Equation (2).

**Table 2:** Comparison of the surface properties of the present FEA coating with previously reported UV-curable fluorinated systems.

System	F (wt%)	θ_a_ (°)	γ_s_^d^ (mJ/m^2^)	γ_s_^nd^ (mJ/m^2^)	γ_s_ (mJ/m^2^)	Ref.
FEA 100 (this work)	31.4	118	19.0	0.2	19.2	-
FPUA H-100	32.8	95.2	19.3	3.4	22.7	[10]
FPUA M-100	28.7	92.0	8.4	10.7	19.1	[10]
TPGDA (non-fluorinated)	0	68.7	6.0	32.9	38.9	[10]

θ_a_ = advancing water contact angle; γ_s_^d^ and γ_s_^nd^ = dispersive and non-dispersive (polar) component of the surface free energy; γ_s_ = total surface free energy. All values were obtained by the Owens–Wendt geometric-mean method using water and ethylene glycol as test liquids. Data for the FPUA and TPGDA films are taken from Ref. [10].

## Data Availability

The data presented in this study are available in the article and Supplementary Materials.

## Supplementary Materials

The following supporting information can be downloaded at https://www.mdpi.com/article/10.3390/polym18182259/s1. Scheme S1: Synthetic Scheme of Fluorinated Epoxy Acrylate (FEA) Oligomer; Table S1: Nomenclature and Chemical Composition of the Materials for Films; Figure S1: ^1^H NMR spectrum (300 MHz, CDCl_3_) of compound 1; Figure S2: ^19^F NMR spectrum (282 MHz, CDCl_3_) of compound 1; Figure S3: ^1^H NMR spectrum (300 MHz, CDCl_3_) of compound 3; Figure S4: ^19^F NMR spectrum (282 MHz, CDCl_3_) of compound 3; Figure S5: FT-IR spectrum of compound 1; Figure S6: FT-IR spectrum of compound 2; Figure S7: FT-IR spectrum of compound 3.

## Abbreviations

The following abbreviations are used in this manuscript:

FEA	Fluorinated epoxy acrylate
FA	Mono-substituted fluorinated alcohol
HFB	Hexafluorobenzene
HDDA-F	2,2,3,3,4,4,5,5-Octafluorohexane-1,6-diyl diacrylate
DGDA	Diglycerol diacrylate
IPDI	Isophorone diisocyanate
DMAc	*N*,*N*-Dimethylacetamide
