# Supplementary material for: Self-Assembling Comb-like Fluorinated Epoxy Acrylates for Low Surface Energy UV-Curable Coatings
Source: Polymers (Basel). 2026 Sep 16;18(18):2259. doi: 10.3390/polym18182259 (PMC13611117; doi:10.3390/polym18182259)
Supplement: Supplementary file 1 [file polymers-18-02259-s001.zip › polymers-4474352-supplementary.pdf]

Supplementary Materials

Self-Assembling Comb-Like Fluorinated Epoxy Acrylates for Low Surface Energy UV-Curable Coatings

Yejung Lee[1], Hyesu Jang[1], Daeun Jeong[2], Su Bin Ji[2], Ye Rim Cho[2], Namhee Kim[1], Myung Hwa Kim[1], and Yong-Ju Kwon[2,*]

[1]*Department of Chemistry, Ewha Womans University, Seoul 03760, Republic of Korea*
[2]*Department of Chemistry, Seoul Women's University, Seoul 01797, Republic of Korea*
yongju_kwon@swu.ac.kr

Table of Contents

1. General Experimental Information S2
2. Materials and Methods S3–S5
3. ^{1}H, and ^{19}F NMR Spectra S6–S9
4. IR Spectra S10-S11

1. General Experimental Information

Unless otherwise stated, all chemical reagents were purchased from commercial suppliers and were used without further purification. Potassium carbonate anhydrous (extra pure grade), isopropyl alcohol (IPA, extra pure grade), and hydrochloric acid (HCl, > 35.0%) were purchased from Duksan. *N*,*N*-dimethylacetamide (DMAc, anhydrous 99.8%), butyl acetate (BA, anhydrous ≥ 99%), 2,2,3,3,4,4,5,5-octafluorohexane-1,6-diol, and hexafluorobenzene (HFB, 99%) were purchased from Sigma-Aldrich. 2,2,3,3,4,4,5,5-Octafluorohexane-1,6-diyl diacrylate (HDDA-F) was purchased from WITHCHEM Co., Ltd. Diglycerol diacrylate (DGDA) and isophorone diisocyanate (IPDI) were provided by URAY Co., Ltd. For the film preparation, Irgacure 184 (5 wt%) was utilized as a photoinitiator. The photopolymerization was carried out for 30 minutes in a UV cube (UVACUBE 400, Dr. Hönle AG, Germany) under a dry Ar gas atmosphere to avoid oxygen inhibition during the curing process. Fourier transform infrared (FT-IR) spectra were obtained using a Scimitar Series FTS800 (Varian) from 32 scans over the range of 380–4000 cm^{-1} at a resolution of 4 cm^{-1}. ^{1}H-NMR and ^{19}F-NMR spectra were recorded on a 300 MHz Bruker NMR spectrometer using $CDCl_3$ as a solvent. The contact angles of water and ethylene glycol were measured using a contact angle meter (CAM100, KSV instruments) at room temperature to investigate the surface energy of the films. The surface morphology of the films was observed using field emission scanning electron microscopy (FE-SEM, JEOL, JSM-6700F). All samples were prepared with a Pt coating to avoid film melting caused by beam damage. Thermal stability was evaluated using a TA Instrument Q600 thermogravimetric analyzer. Samples were scanned from room temperature to 550 °C at a heating rate of 10 °C/min under a nitrogen purge of 100 mL/min. To study the photopolymerization under UV-radiation, photo differential scanning calorimetry (photo-DSC) was conducted under 10 mW/cm^2 for 3 minutes at room temperature. Nitrogen gas was purged at a rate of 50 mL/min to prevent oxygen inhibition.

2. Materials and Methods

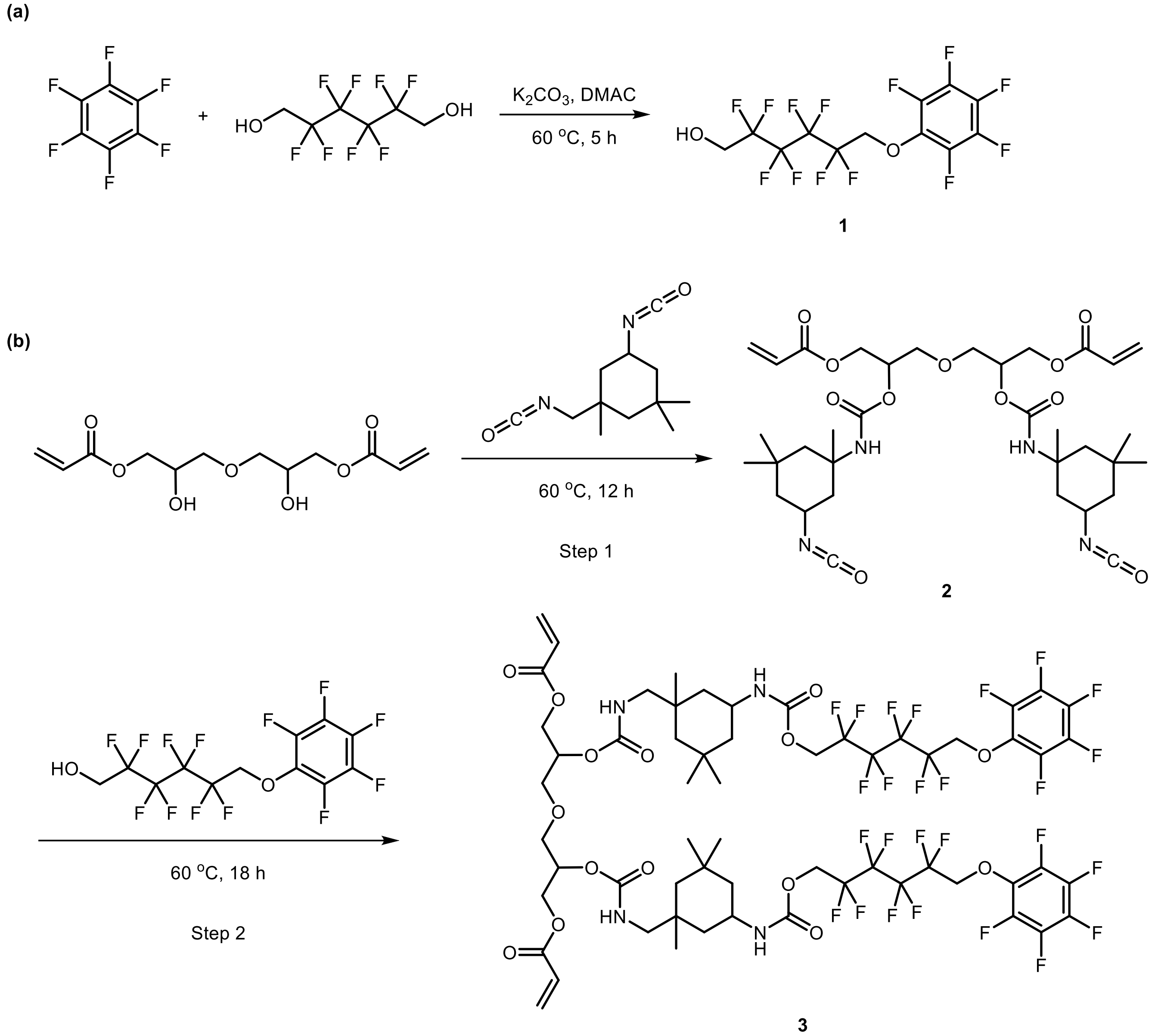

Scheme S1. Synthetic Scheme of Fluorinated Epoxy Acrylate (FEA) Oligomer

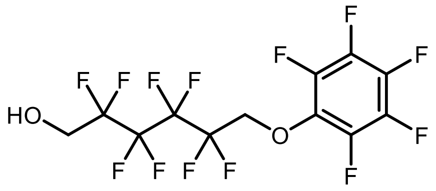

1

2,2,3,3,4,4,5,5-Octafluoro-6-(perfluorophenoxy)hexan-1-ol (1)

The reaction was carried out in a 100 mL three-neck round-bottom flask equipped with a dropping funnel, a nitrogen inlet, a water condenser, and a thermometer. Under a nitrogen atmosphere, hexafluorobenzene (14.2 g, 76.30 mmol, 2.0 equiv), anhydrous potassium carbonate (6.86 g, 49.60 mmol, 1.3 equiv), and 2,2,3,3,4,4,5,5-octafluorohexane-1,6-diol (10.0 g, 38.15 mmol, 1.0 equiv) were reacted in anhydrous DMAc (60 mL) at 60 °C for 5 h. An excess of hexafluorobenzene was employed to suppress the di-substitution of both hydroxyl groups of the diol. After the reaction, a 3% aqueous HCl solution was added dropwise with vigorous stirring until the mixture became acidic (pH < 2), as confirmed by pH test paper. The mixture was transferred to a separatory funnel and washed four times with distilled water until the aqueous layer was neutralized, and the organic phase was collected and concentrated under reduced pressure. The crude product, which contained a small amount of the di-substituted byproduct, was purified by flash column chromatography on silica gel (hexane/ethyl acetate = 4:1, v/v) to afford the title compound as a colorless oil (6.5 g, 40%). ^{1}**H NMR** (300 MHz, $CDCl_3$) δ 4.71–4.47 (m, 2H), 4.26–3.95 (m, 2H); the OH proton was not observed, presumably due to exchange broadening. ^{19}**F{**^{1}**H} NMR** (282 MHz, $CDCl_3$) δ -121.4 (dddt, *J* = 14.8, 12.4, 6.2, and 3.3 Hz, 2F), -122.2–-123.5 (m, 2F), -123.5–-125.4 (m, 4F), -155.3–-156.7 (m, 2F), -160.4–-161.7 (m, 1F), -161.7–-163.1 (m, 2F).

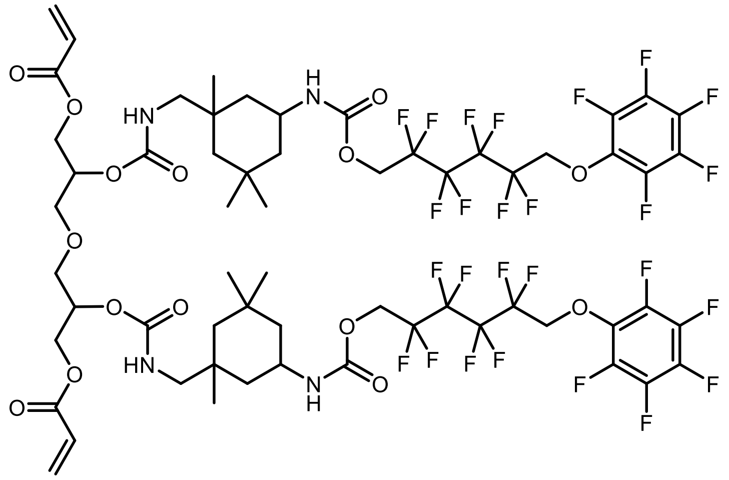

3

Oxybis(2-((((1,3,3-trimethyl-5-((((2,2,3,3,4,4,5,5-octafluoro-6-(perfluorophenoxy)hexyl)oxy)carbonyl)-amino)cyclohexyl)methyl)carbamoyl)oxy)propane-3,1-diyl) diacrylate (3)

Under a nitrogen atmosphere, oxybis(2-hydroxypropane-3,1-diyl) diacrylate (1.37 g, 5 mmol) and butyl acetate (10 mL) were added to a 3-neck round-bottom flask. The flask was placed in an oil bath and heated

to 60 °C, after which IPDI (2.23 g, 10 mmol) was added dropwise. The reaction mixture was stirred and maintained at 60 °C for 12 h to obtain oxybis(2-((((5-isocyanato-1,3,3-trimethylcyclohexyl)methyl)-carbamoyl)oxy)propane-3,1-diyl) diacrylate **2**. Under a nitrogen atmosphere, the intermediate oxybis(2-((((5-isocyanato-1,3,3-trimethylcyclohexyl)methyl)carbamoyl)oxy)propane-3,1-diyl) diacrylate obtained in Step 1 was heated to 60 °C in an oil bath. Subsequently, 2,2,3,3,4,4,5,5-octafluoro-6-(perfluoro-phenoxy)hexan-1-ol (4.29 g, 10 mmol) was added dropwise. The reaction mixture was stirred at 60 °C for 18 h and then cooled to room temperature. Isopropyl alcohol (IPA, 10 mL) was added to the solution to quench the reaction. Finally, the reaction mixture was concentrated under reduced pressure using a rotary evaporator to afford oxybis(2-((((1,3,3-trimethyl-5-((((2,2,3,3,4,4,5,5-octafluoro-6-(perfluorophenoxy)-hexyl)oxy)carbonyl)amino)cyclohexyl)methyl)-carbamoyl)oxy)propane-3,1-diyl) diacrylate **3** (4.26 g, 54%). **^{1}H NMR** (300 MHz, $CDCl_3$) δ 6.40 (dd, *J* = 17.3 and 5.4 Hz, 2H), 6.10 (dd, *J* = 17.3 and 10.4 Hz, 2H), 5.84 (d, *J* = 10.3 Hz, 2H), 5.35–4.77 (m, 4H), 4.57 (t, *J* = 13.2 Hz, 2H), 4.33 (d, *J* = 35.3 Hz, 4H), 4.11–3.94 (m, 2H), 3.89–3.50 (m, 6H), 3.35–2.54 (m, 5H), 1.68 (s, 5H), 1.30–1.10 (m, 4H), 1.10–0.61 (m, 26H); **^{19}F{^{1}H} NMR** (282 MHz, $CDCl_3$) δ -120.1–-120.9 (m, 2F), -121.1–-122.0 (m, 4F), -122.2–-123.0 (m, 2F), -123.6–-124.7 (m, 8F), -156.4 (d, *J* = 25.3 Hz, 4F), -160.9 – -161.8 (m, 2F), -162.2–-163.2 (m, 4F).

Table S1. Nomenclature and Chemical Composition of the Materials for Films

Designation	Content (wt%)		
	FEA	HDDA-F	Fluorine
FEA 100	100	0	31.4
FEA 70	70	30	34.3
FEA 50	50	50	36.3
FEA 30	30	70	38.2
FEA 0	0	100	41.1

3. ^{1}H, and ^{19}F NMR Spectra

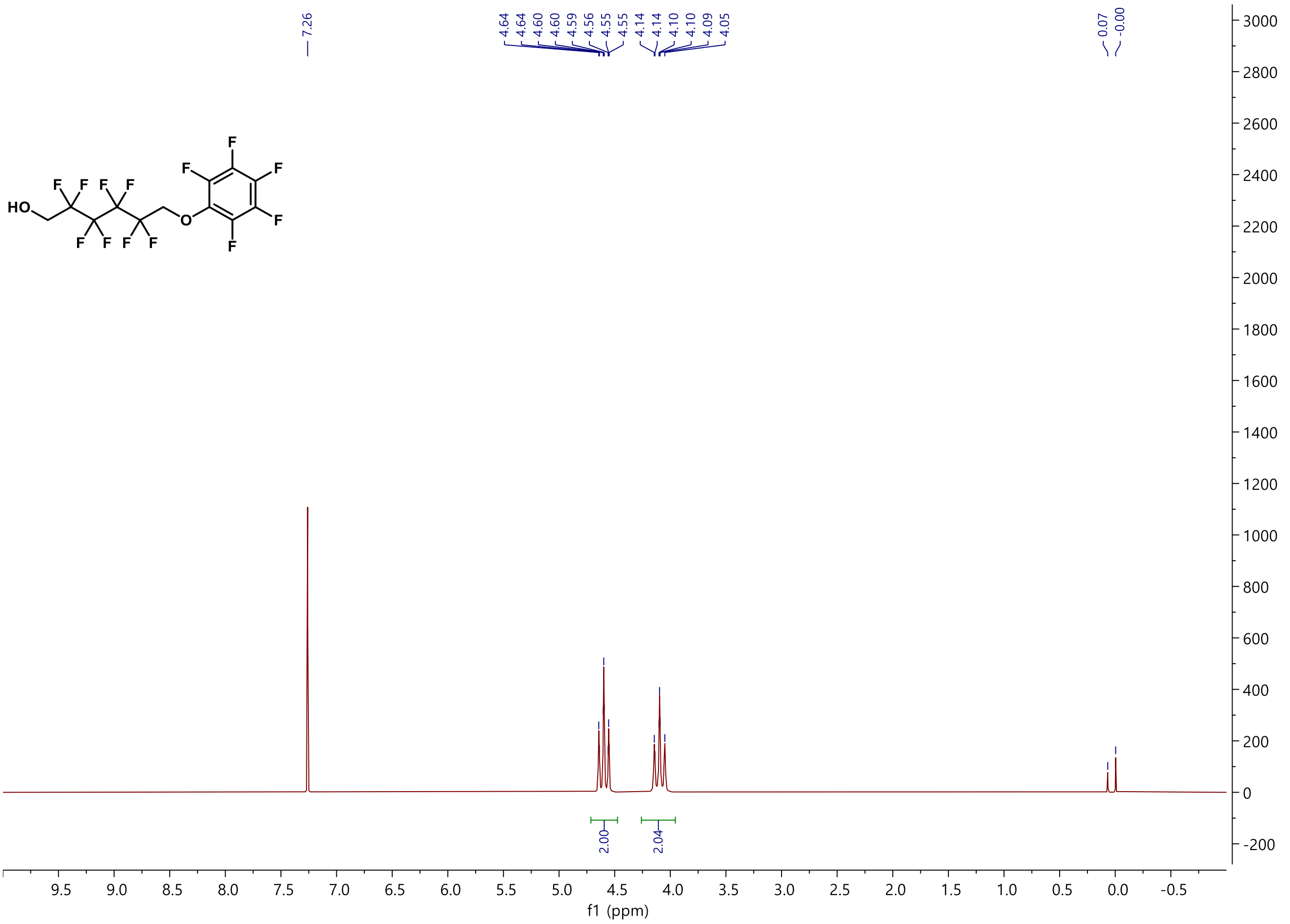

Figure S1. ^{1}H NMR spectrum (300 MHz, $CDCl_3$) of compound **1**.

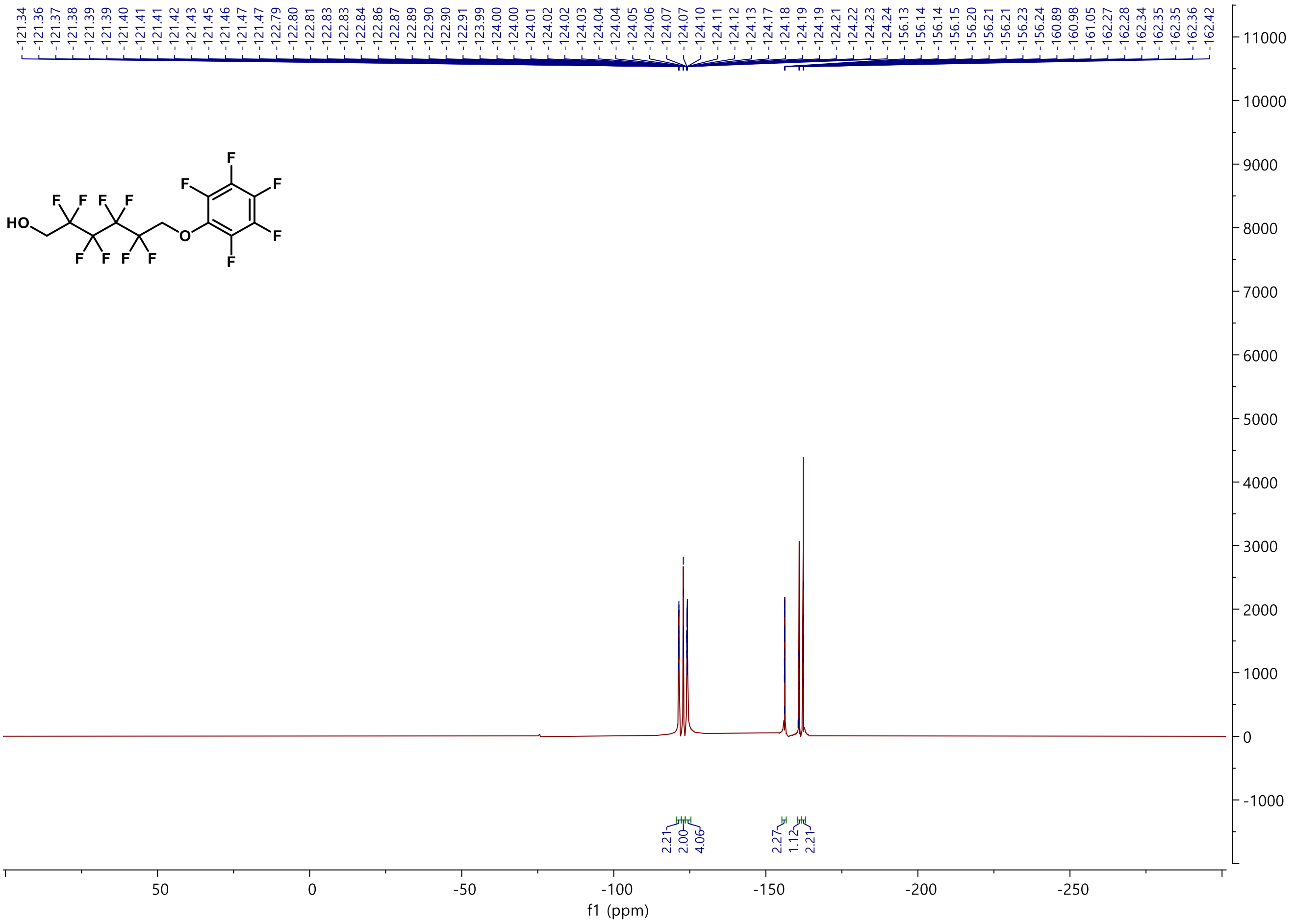

Figure S2. ^{19}F NMR spectrum (282 MHz, $CDCl_3$) of compound **1**.

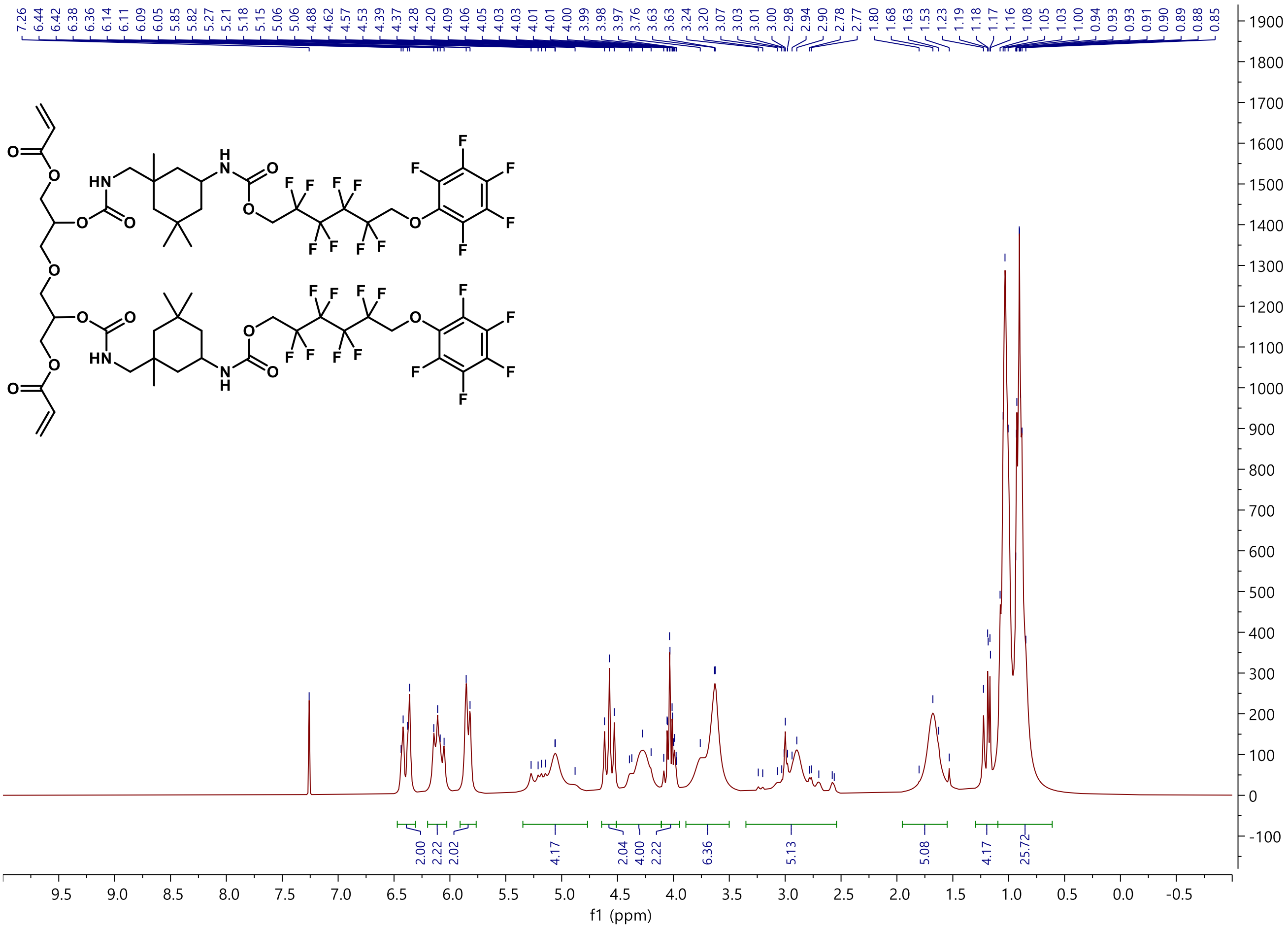

Figure S3. ^{1}H NMR spectrum (300 MHz, $CDCl_3$) of compound **3**.

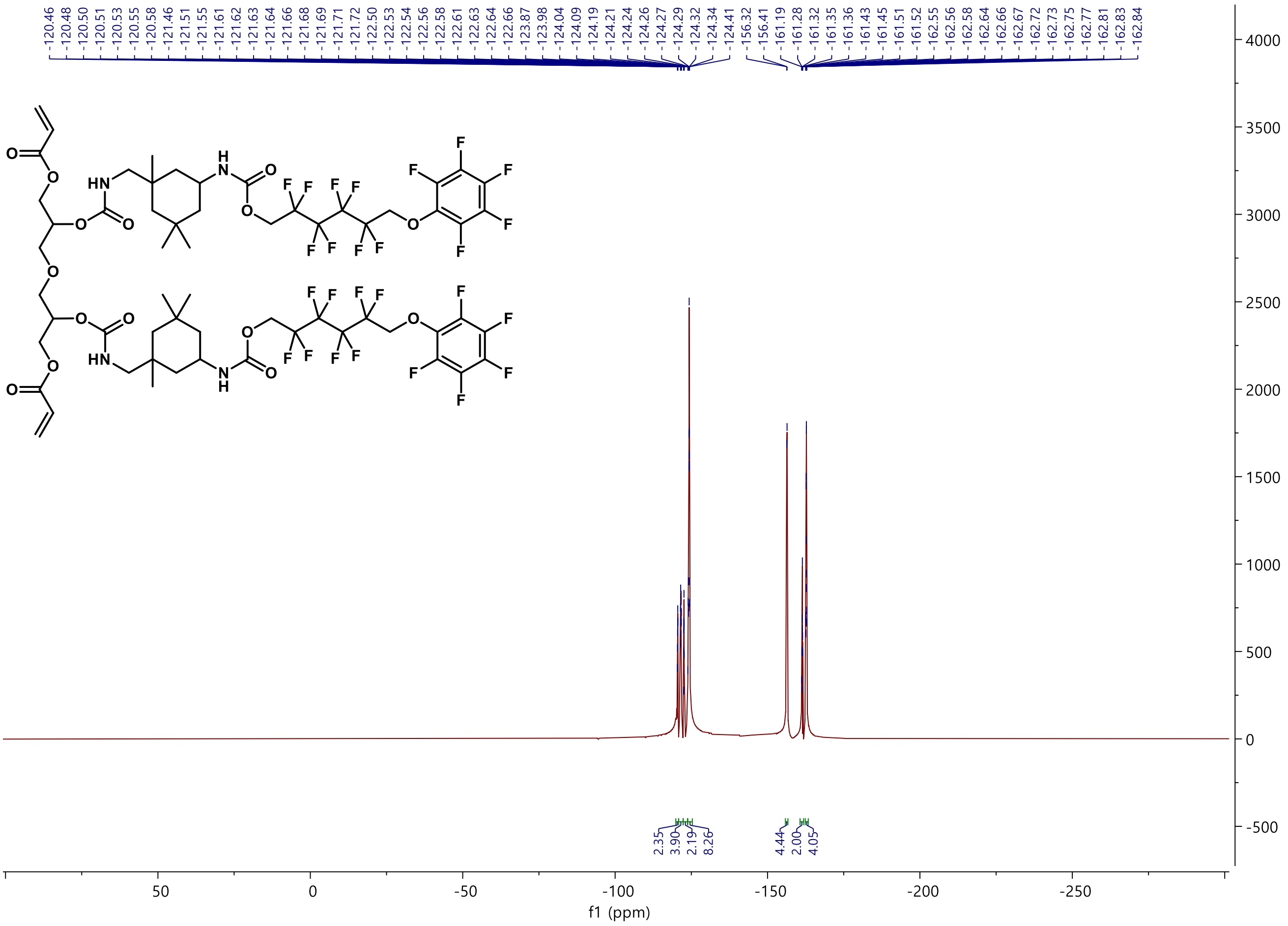

Figure S4. ^{19}F NMR spectrum (282 MHz, $CDCl_3$) of compound **3**.

4. IR Spectra

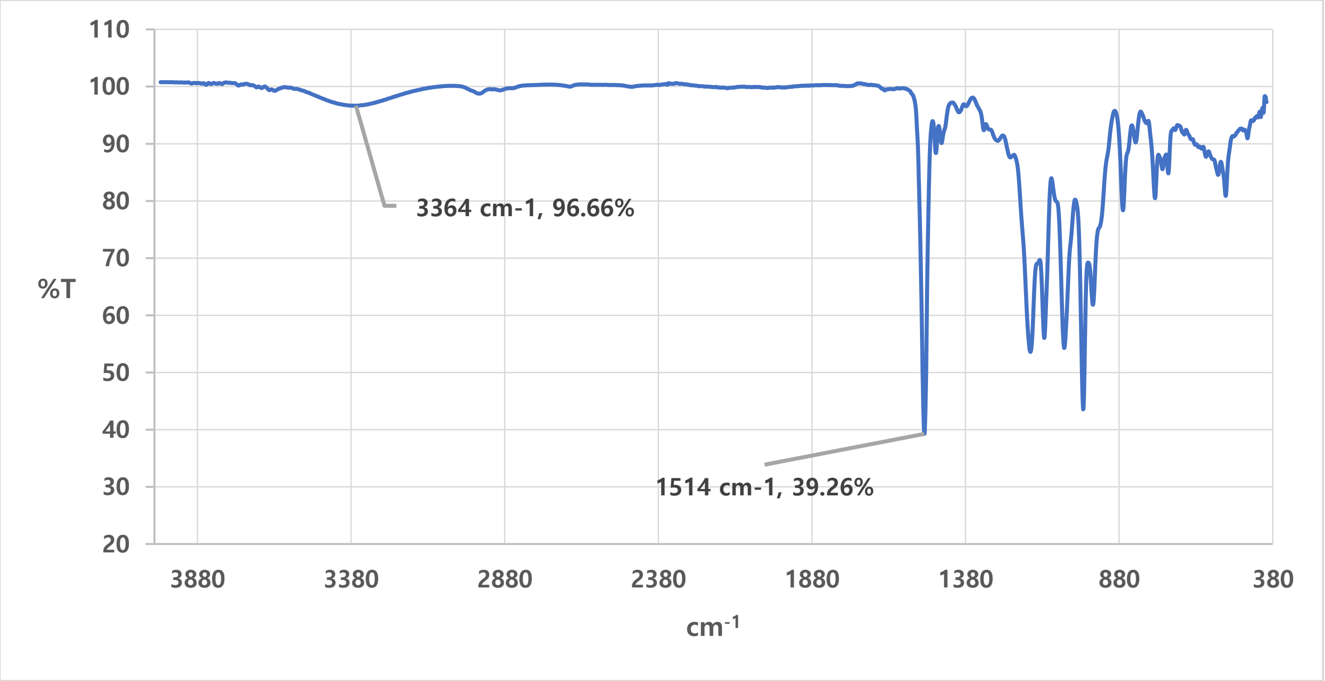

Figure S5. FT-IR spectrum of compound **1**.

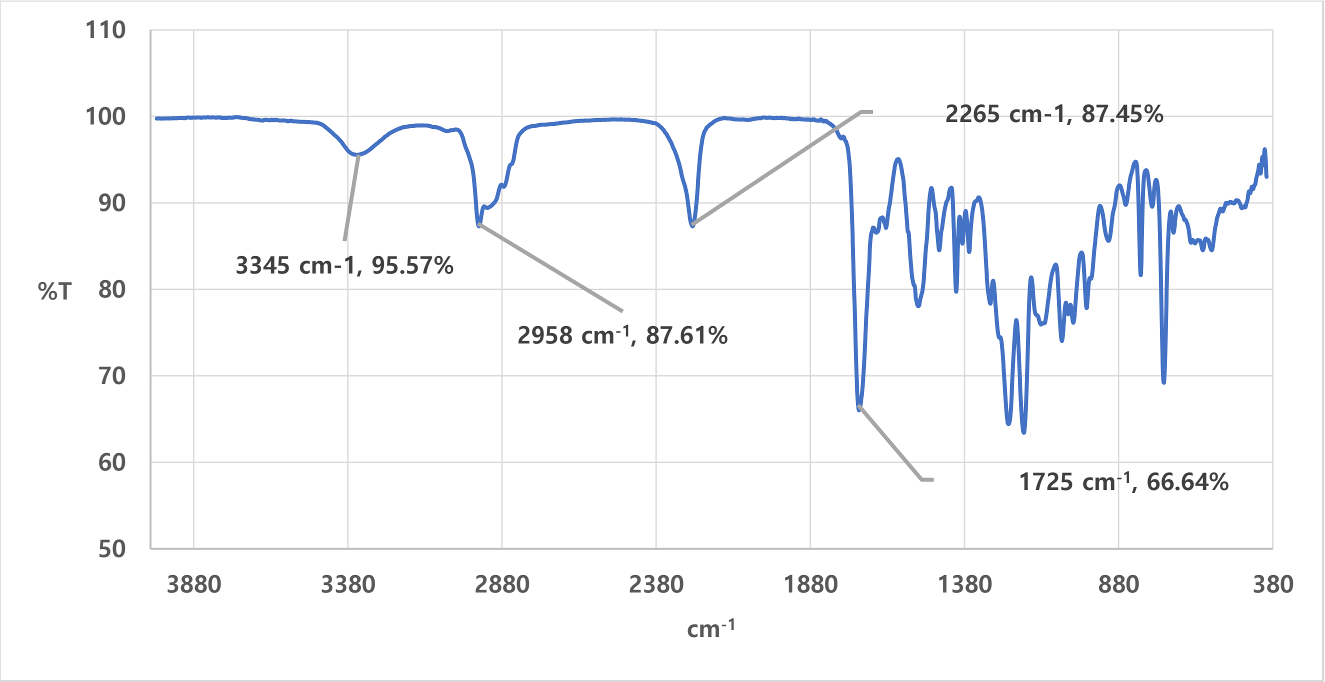

Figure S6. FT-IR spectrum of compound **2**.

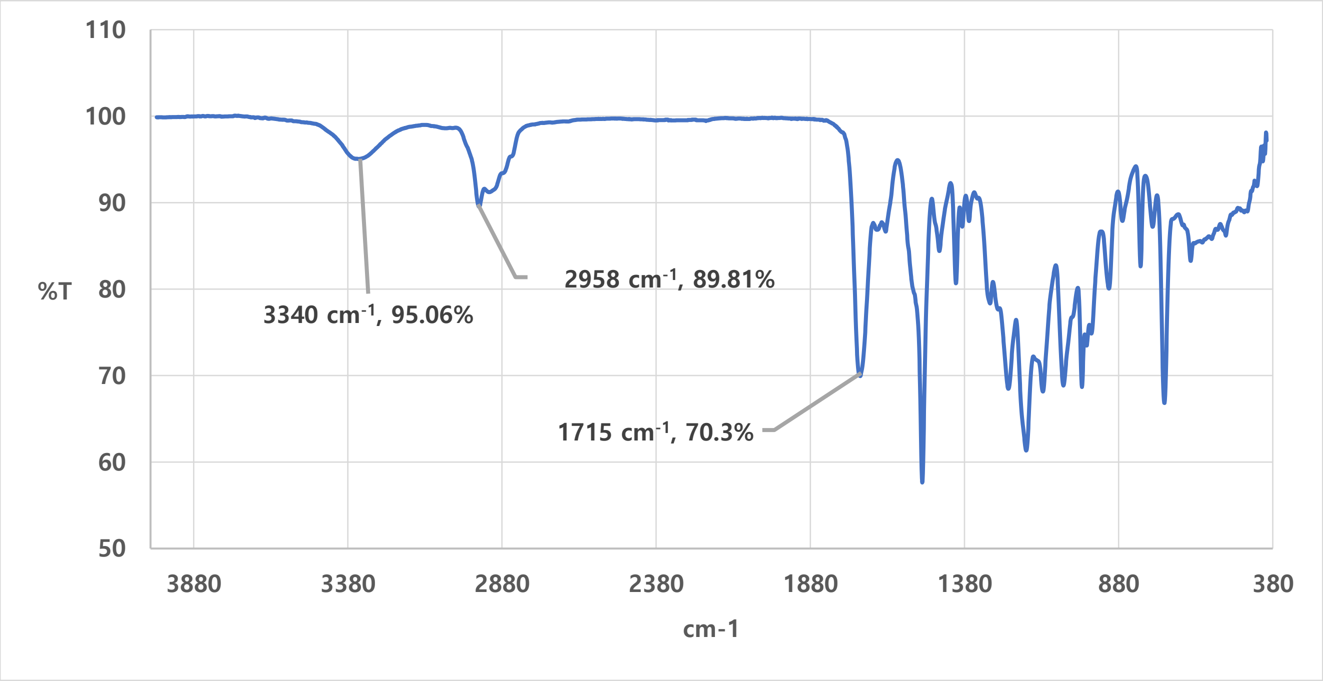

Figure S7. FT-IR spectrum of compound **3**.
